# Evaluation of the potential of Rejuveinix plus dexamethasone against sepsis

**DOI:** 10.2217/fmb-2022-0044

**Published:** 2022-09-02

**Authors:** Fatih M Uckun, Muhammad Saeed, Mustafa Awili, Ibrahim H Ozercan, Sanjive Qazi, Cynthia Lee, Adeel Shibli, Alan W Skolnick, Alonso Prusmack, Joseph Varon, Cesar IP Barrera, Cemal Orhan, Michael Volk, Kazim Sahin

**Affiliations:** ^1^Drug Discovery Program, Reven Pharmaceuticals, Westminster, CO 80234, USA; ^2^Department of Developmental Therapeutics, Immunology & Integrative Medicine, Ares Pharmaceuticals, St Paul, MN 55110, USA; ^3^PRX Research & Dallas Regional Medical Center, Dallas, TX 75149, USA; ^4^Department of Pathology Faculty of Medicine, Firat University, Elazig, 23119, Turkey; ^5^Memorial Hermann Memorial City Medical Center, Houston, TX 77024, USA; HD Research, Bellaire, TX 77401, USA; ^6^United Memorial Medical Center, Houston, TX 77091, USA; ^7^Department of Animal Nutrition, Faculty of Veterinary, Firat University, Elazig, 23119, Turkey

**Keywords:** COVID-19, inflammation, LPS, RJX, sepsis, vitamins

## Abstract

**Aim:** Our main objectives were to compare the effects of Rejuveinix (RJX), dexamethasone (DEX) and their combination on the severity of sepsis and survival outcome in an animal model of fatal sepsis. **Methods:** We used the LPS plus D-galactosamine mouse model of sepsis to compare the anti-inflammatory activities of RJX, dexamethasone and a combination of RJX plus DEX. Additionally, we examined the clinical feasibility and tolerability of combining RJX with DEX in COVID-19 patients in a clinical phase I study. Data were analyzed using standard methods. **Results & conclusion:** RJX exhibited potent anti-inflammatory activity in the murine sepsis model. The combination of RJX plus DEX was more effective than either agent alone, decreased the inflammatory cytokine responses and associated organ damage, and improved the survival outcome in mice. In the phase I clinical study, RJX plus DEX was well tolerated by COVID-19 patients.

Sepsis represents a strong systemic inflammatory response to an infection with a potentially fatal outcome due to its complications [1–9]. Severe viral sepsis caused by SARS-CoV-2 shows a rapid progression associated with cytokine release syndrome and a high case fatality rate in high-risk COVID-19 patients [10–16]. Our antisepsis drug candidate, Rejuveinix (RJX), comprises several anti-inflammatory and antioxidant vitamins [17–28]. It has exhibited promising single-agent anti-inflammatory activity in mice challenged with LPS plus D-galactosamine (LPS-GalN) [17]. Specifically, it decreased the inflammatory cytokine responses and improved survival when administered prophylactically in mice challenged with LPS-GalN. Furthermore, preliminary experiments have suggested that it may also be effective in reversing sepsis if administered after the onset of sepsis [17,19]. In addition, RJX showed a favorable clinical safety and pharmacokinetics profile in a recently completed randomized, double-blind, placebo-controlled phase I ascending dose-escalation study in healthy volunteers (Clinical Trial Registration: NCT03680105 [ClinicalTrials.gov]) [17].

The primary objective of the present study was to compare the effects of RJX, the standard anti-inflammatory drug dexamethasone (DEX) and their combination on the severity of sepsis and survival outcome in an animal model that employed LPS-GalN to induce fatal sepsis. We hypothesized that RJX, especially when combined with DEX, would improve the survival outcome of mice developing systemic inflammation after injection with a lethal dose of LPS-GalN [23]. Here, we first confirm and extend our previous study of the single-agent activity of RJX in mice challenged with LPS-GalN [17,23]. We demonstrate that RJX – at a dose level >ten-times lower than its clinical maximum tolerated dose (MTD) – exhibits potent single-agent anti-inflammatory activity and is as effective as DEX at standard dose levels in this model of fatal sepsis [23]. The combination of RJX plus DEX immediately and profoundly decreased the inflammatory cytokine (IL-6, TNF-α) responses to LPS-GalN, mitigated the inflammatory tissue damage in the lungs and liver, and prevented a fatal outcome [23]. Although the treatments were started after the onset of fulminant cytokine storm characterized by markedly elevated serum IL-6 and TNF-α levels and systemic inflammation, as well as severe lung damage, a near-complete recovery of the inflammatory lung injury was achieved within 24 h and the survival outcome was improved [23]. We also report data from an early-stage clinical evaluation of RJX in COVID-19 patients with systemic inflammation.

## Materials & methods

### Rejuveinix

RJX is a formulation that contains multiple antioxidant vitamins with anti-inflammatory properties [17,21].

### LPS-GalN model of fatal cytokine storm & sepsis

The RJX doses were 0.7 ml/kg (∼14 μl/20 g mouse) and 1.4 ml/kg (∼28 μl/20 g/mouse), and they were administered as intraperitoneal (ip.) bolus injections. The doses of the active ingredients at the 0.7 ml/kg dose were 31.5 mg/kg vitamin C, 2.2 mg/kg vitamin B1, 0.09 mg/kg vitamin B2, 4.2 mg/kg vitamin B3, 0.1 mg/kg vitamin B5, 4.2 mg/kg vitamin B6, 0.07 mg/kg vitamin B12 and 28.3 mg/kg magnesium sulfate. The doses for the active ingredients at the 1.4 ml/kg dose were 63 mg/kg vitamin C, 4.4 mg/kg vitamin B1, 0.18 mg/kg vitamin B2, 8.4 mg/kg vitamin B3, 0.2 mg/kg vitamin B5, 8.4 mg/kg vitamin B6, 0.14 mg/kg vitamin B12 and 56.6 mg/kg magnesium sulfate [17]. These sub-MTD dose levels were based on the observation of single-agent activity in the LPS-GalN mouse model of sepsis [17]. The 0.7 ml/kg mouse dose corresponds to a human equivalent dose (HED) of 0.057 ml/kg of RJX, which is 7.5% of its MTD of 0.759 ml/kg determined in the previously published randomized phase I clinical trial in healthy volunteers [17]. The 1.4 ml per kg mouse dose corresponds to HED of 0.114 ml/kg of RJX, which is 15% of its MTD [23].

In the reported experiments, the Guide for the Care and Use of Laboratory Animals was followed. The study received approval from the Animal Care and Use Committee of Firat University (no. 420629). Male BALB/c mice (6–8 weeks old and weighing on average 20 g), which were purchased from Laboratory Animal Center of Firat University, were randomly divided into different treatment groups, as previously reported [17]. In order to induce fatal cytokine release syndrome as well as acute lung and liver injury, mice were challenged with an ip. injection of LPS plus D-galactosamine (Sigma, MO, USA). Each mouse received a 500 μl ip. injection of LPS-GalN (consisting of 100 ng of LPS plus 8 mg of D-galactosamine) [23]. Treatments were delayed until 2 h post-LPS-GalN injection when mice had a fulminant systemic inflammation with very severe lung and liver damage, as well as markedly elevated inflammatory cytokine levels [23]. Vehicle control mice were treated with 0.5 ml normal saline (NS) instead of RJX. NS was administered ip. 2 h after LPS-GalN. Test mice received either RJX (0.7 ml/kg or 1.4 ml/kg) or DEX (0.1 mg/kg, 0.6 mg/kg or 6.0 mg/kg) as a monotherapy in a side-by-side comparison. We also examined a combination of 0.7 ml/kg RJX with either 0.6 mg/kg or 6 mg/kg DEX at 2 h post-LPS-GalN injection [23]. Drugs were administered ip. in a total volume of 0.5 ml. Tissue levels of oxidative stress biomarkers superoxide dismutase (SOD), catalase and glutathione peroxidase (GSH-Px), ascorbic acid levels and malondialdehyde were determined to examine the effects of RJX, DEX and RJX + DEX on the sepsis-associated oxidative stress. Levels of IL-6 and TNF-α in serum samples were measured by quantitative ELISA using the commercially available Quantikine ELISA kits, as previously reported [17]. At the time of death, blood samples were obtained for biomarker studies. In addition, lungs and liver specimens were subjected to histopathologic examination, as described previously [17].

### Statistical analysis

Standard statistical methods were used in analyzing the experimental data. The Kaplan–Meier method was applied to the analysis of the survival outcome data within the limitations of a small sample size, as reported [17].

### Clinical phase I study

The study examined the safety of RJX plus institutional standard of care (SOC) in hospitalized patients with COVID-19. Patients received daily 40 min (±10 min) infusions of 20 ml RJX mixed with 100 ml normal saline (total volume = 120 ml) intravenously plus SOC for 7 consecutive days. The details, including the statistical analysis of the clinical data, are provided in the Supplementary Data. The study was performed under IND149585 at the following four centers: 1) Memorial Hermann Memorial City Medical Center, TX, USA; 2) Christus Health Santa Rosa Hospital, TX, USA; 3) LFROS Research and United Memorial Medical Center, TX, USA; and 4) PRX Research and Dallas Regional Medical Center, TX, USA.

## Results

### Effectiveness of treatment with RJX in a side-by-side comparison to DEX in reversing acute lung & liver injury & improving survival in mice injected with LPS-GalN

LPS-GalN-challenged mice experience a rapid onset systemic inflammation with markedly elevated inflammatory cytokine levels as well as severe lung injury, as early as 2 h after the administration of LPS-GalN [17]. We first set out to determine the effects of RJX versus DEX on the LPS-GalN-induced inflammatory cytokine response in BALB/c mice [23] (Supplementary Figure 1). At 2 h post-injection of LPS-GalN, the serum IL-6 and TNF-α levels were markedly elevated in electively terminated control mice consistent with an inflammatory cytokine response. A single low-dose RJX at the assigned low dose levels of 0.7 ml/kg (HED: 0.057 ml/kg; 7.5% of human MTD) and 1.4 ml/kg (HED: 0.114 ml/kg; 15% of human MTD), was administered at 2 h after LPS-GalN injection, and effectively reversed the LPS-GalN-induced increased serum levels of the pro-inflammatory cytokines IL-6 and TNF-α within 24 h [23] (Supplementary Figure 1). A 1.4 ml/kg low-dose (15% of MTD) RJX was significantly more effective than a single DEX injection at the standard 0.6 mg/kg dose level (HED: 0.05 mg/kg; 4 mg standard dose for an 80 kg person) and comparable to the 6.0 mg/kg high-dose DEX (HED: 0.49 mg/kg; 39 mg dose for an 80 kg person, which is higher than the standard 4–8 mg dose levels for DEX) (Supplementary Figure 1). The observed reversal of the inflammatory cytokine response by RJX (0.7 or 1.4 ml/kg) or DEX (0.6 or 6.0 mg/kg) was associated with a partial reversal of the lung and liver injury that was documented at 2 h post-LPS-GalN injection when treatments were initiated (Supplementary Figure 2–4). Specifically, as evidenced in Supplementary Figures 2A & 3, RJX at 0.7 ml/kg (mean ± standard error [SE] acute lung injury [ALI] score: 2.7 ± 0.2) or 1.4 ml/kg (mean ± SE ALI score: 2.3 ± 0.2) low dose levels as well as DEX at the 0.6 mg/kg (HED: 0.05 mg/kg; 4 mg standard dose for an 80 kg person) (mean ± SE ALI score: 2.8 ± 0.3) dose level (but not DEX at 0.1 mg/kg dose level; mean ± SE ALI score: 3.5 ± 0.2) were capable of partially reversing the lung injury that was documented at 2 h post-LPS-GalN injection when treatments were initiated (mean ± SE ALI score: 3.0 ± 0.3), as measured by the lung histopathological scores (i.e., ALI scores) [23]. The best results were obtained with a 6.0 mg/kg supratherapeutic high-dose DEX (HED: 0.49 mg/kg; 39 mg dose for an 80 kg person) (mean ± SE ALI score: 1.8 ± 0.3) (Supplementary Figure 3A). By comparison, the lung damage further progressed in control mice treated with NS (vehicle) (mean ± SE ALI score: 3.7 ± 01) [23]. Similar to its effects on the LPS-GalN-induced ALI, 0.7 ml/kg or 1.4 ml/kg low-dose RJX, as well as standard 0.6 mg/kg and very high 6.0 mg/kg dose levels of DEX (but not DEX at 0.1 mg/kg dose level), significantly reduced the liver injury (Supplementary Figures 2 & 4). The histopathological liver damage scores (mean ± SE) were 0 ± 0 for control mice not challenged with LPS-GalN, 3.5 ± 0.3 for mice electively terminated 2 h post LPS-GalN, 3.5 ± 0.2 for mice treated with NS post LPS-GalN, 3.3 ± 0.2 for 0.1 mg/kg DEX, 2.7 ± 0.2 for 0.6 mg/kg DEX, 2.3 ± 0.2 for 6.0 mg/kg DEX, 2.5 ± 0.2 for 0.7 ml/kg RJX and 2.3 ± 0.2 for 1.4 ml/kg RJX [23].

RJX treatment significantly improved the survival of mice (Supplementary Figure 5). By comparison, treatment with NS included as vehicle control did not reverse the fulminant cytokine response or prevent its progression. Notably, 0.7 ml/kg low-dose RJX was moderately more effective than DEX at a 0.1 mg/kg low dose level (median survival: 15.1 h vs 5.1 h; 24-h mortality: 50% vs 83.3%), and it was as effective as DEX at the standard dose 0.6 mg/kg (Supplementary Figure 5). Notably, at a dose level of 1.4 ml/kg, which corresponds to 15% of its clinical MTD, RJX reduced the mortality to 40% (median survival >24 h). These results were very similar to and statistically not different from the 33.3% mortality (median survival >24 h; p = 0.99) achieved with DEX at the supratherapeutic 6.0 mg/kg dose level that is 4.9–9.8-fold higher than the standard 4–8 mg clinically applied dose levels for DEX [23] (Supplementary Figure 5).

### Effectiveness of treatment with RJX in combination with DEX in reversing fatal cytokine storm, acute lung & liver injury & improving survival in mice injected with LPS-GalN

**Figure 1.**
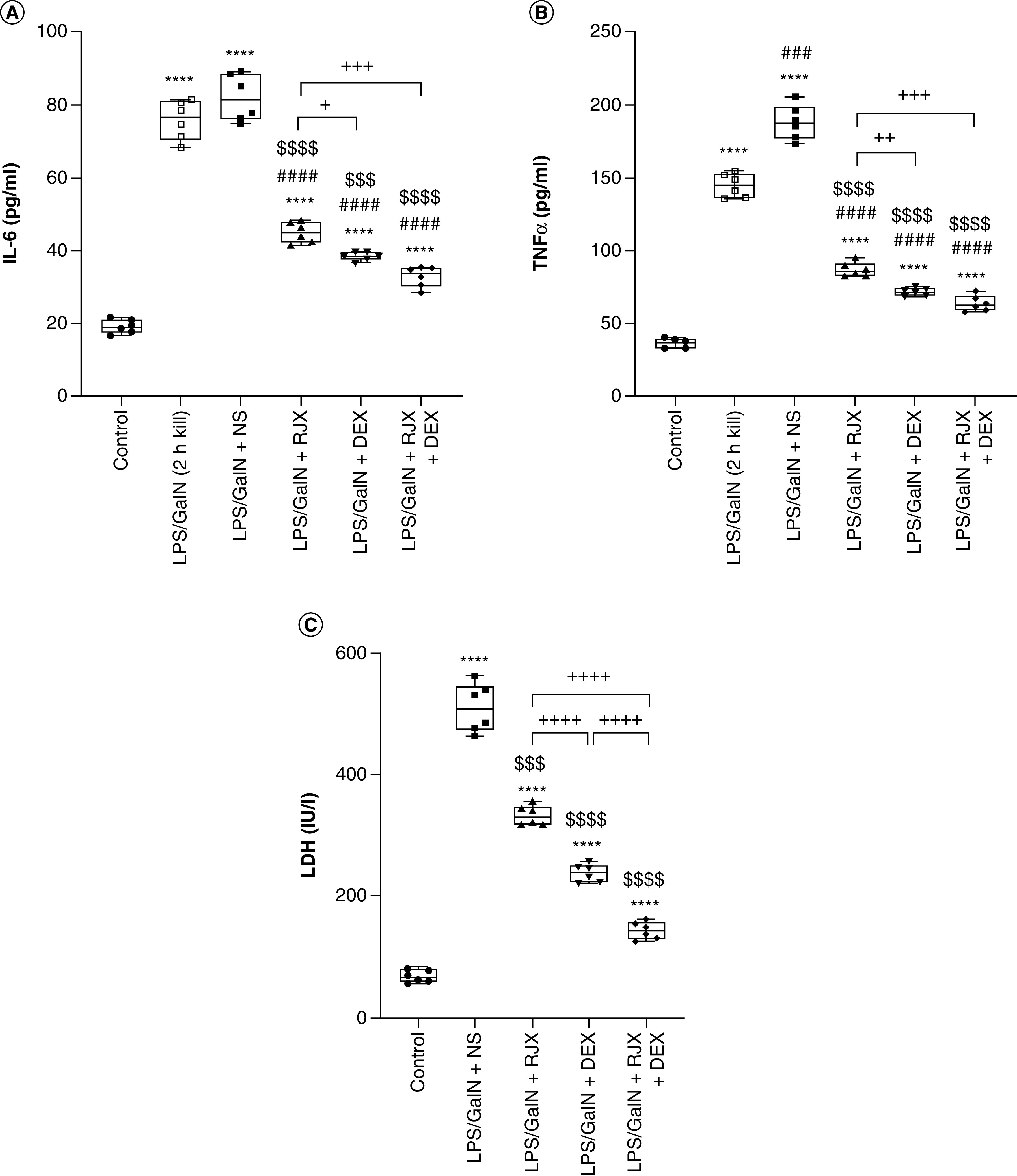
Therapeutic use of low-dose RJX plus supratherapeutic high-dose DEX combination after onset of systemic inflammation and lung injury reverses inflammatory cytokine response and systemic inflammation in the LPS-GalN mouse model of fatal cytokine storm and sepsis. Depicted are the effects of RJX, DEX and RJX + DEX combination treatments on serum levels of IL-6 **(A)**, TNF-α **(B)** and LDH **(C)** [23]. Groups of six BALB/c mice were treated with intraperitoneal injections of RJX (sixfold diluted, 4.2 ml/kg, 0.5 ml/mouse), DEX (6 mg/kg, 0.5 ml/mouse), RJX + DEX (0.5 ml/mouse) or vehicle (NS, 0.5 ml/mouse) 2 h post-injection of LPS-GalN. Except for untreated control mice (control), each mouse received 0.5 ml of LPS-GalN (consisting of 100 ng of LPS plus 8 mg of D-galactosamine) intraperitoneally. The depicted whisker plots represent the median and values. Welch's analysis of variance and Tamhane's T2 *post hoc* test were used for comparing the results among different treatment groups. Statistical significance between groups is shown by ****p < 0.0001 as compared with control group; ^###^p < 0.001; ^####^p < 0.0001 as compared with LPS/GaIN (2 h kill) group; ^$$$^p < 0.001; ^$$$$^p < 0.0001 as compared with LPS/GaIN + NS and ^+^p < 0.05; ^++^p < 0.01; ^+++^p < 0.001; ^++++^p < 0.0001 pairwise comparisons between the groups. DEX: Dexamethasone; GalN: Galactosamine; LDH: Lactate dehydrogenase; LPS: Lipopolysaccharide; NS: Normal saline; RJX: Rejuveinix.

Low-dose RJX (0.7 ml/kg) plus high-dose DEX (6.0 mg/kg) effectively reversed the increased serum levels of the systemic inflammation markers (IL-6, TNF-α and lactate dehydrogenase) within 24 h, and it appeared to be overall more effective than DEX alone or RJX alone (Figure 1) [23]. The serum levels of lactate dehydrogenase, a biomarker of systemic inflammation and tissue damage, were significantly lower in mice treated with the RJX + DEX combination than mice treated with RJX alone (p < 0.0001) or DEX alone (p < 0.0001) (Figure 1).

**Figure 2.**
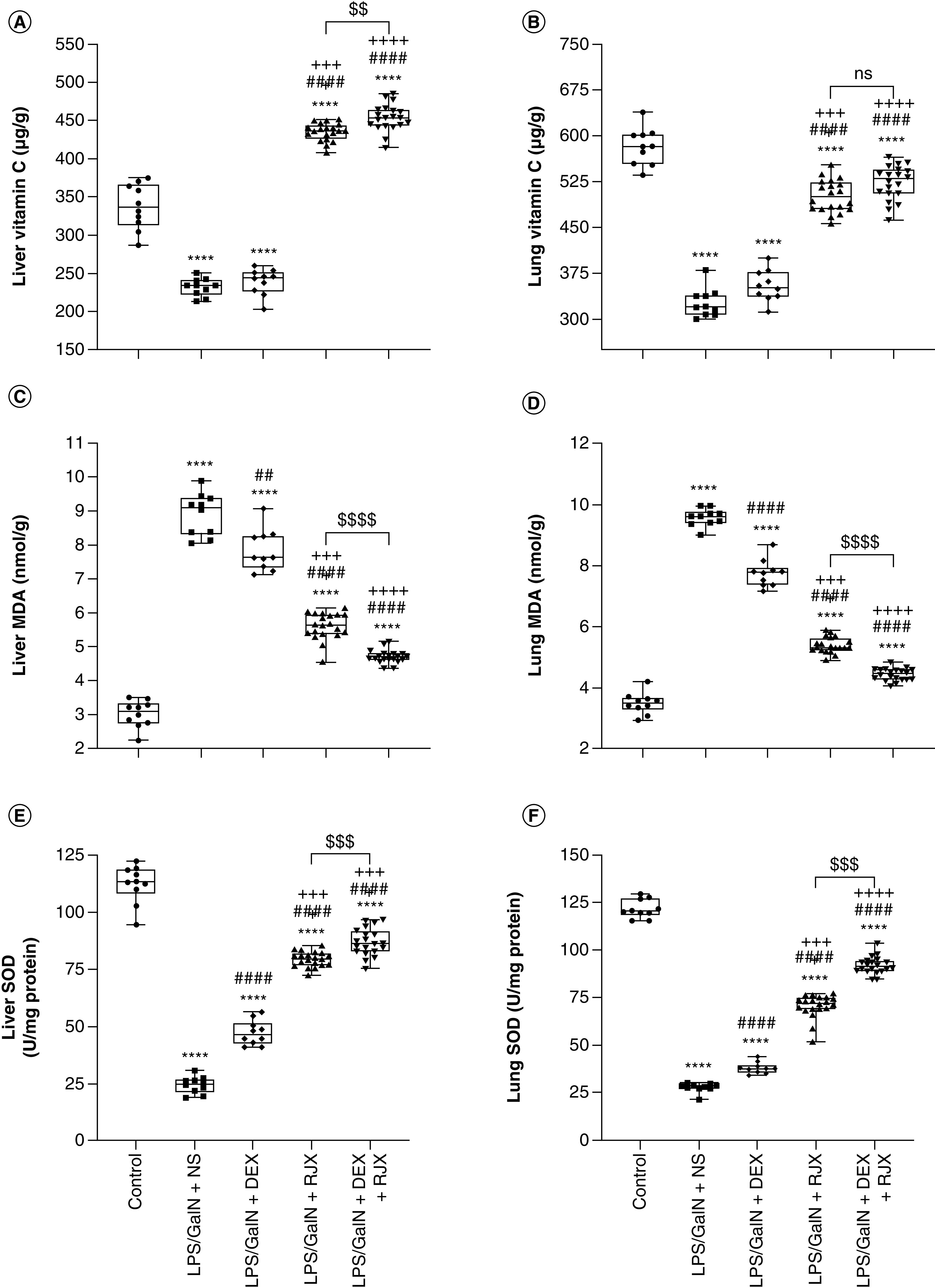
Tissue-level *in vivo* antioxidant activity of RJX, DEX and RJX + DEX in a mouse model of fatal cytokine storm, sepsis, systemic inflammation, ARDS and multiorgan failure. BALB/c mice were treated with intraperitoneal injections of RJX (n = 20, sixfold diluted, 4.2 ml/kg, 0.5 ml/mouse), DEX (n = 10, 6 mg/kg, 0.5 ml/mouse), RJX + DEX (n = 20, 0.5 ml/mouse) or vehicle (NS, 0.5 ml/mouse) 2 h post-injection of LPS-GalN. We used previously published methods and statistical analysis tools [17,21]. Except for untreated control mice (control, n = 10), each mouse received 0.5 ml of LPS-GalN (consisting of 100 ng of LPS plus 8 mg of D-galactosamine) intraperitoneally. The depicted whisker plots represent the median and values. **(A, C, E & F)**, Welch's analysis of variance and Games–Howell *post hoc* test were used for comparing the results among different treatment groups. **(B & D)** Analysis of variance and Tukey's *post hoc* test were used for comparing the results among different treatment groups. Statistical significance between groups is shown by ****p < 0.0001 as compared with control group; ^##^p < 0.01; ^####^p < 0.0001 as compared with LPS/GaIN + NS group;; ^++++^p < 0.0001 as compared with LPS/GaIN + DEX group and ^$$^p < 0.01; ^$$$^p < 0.001; ^$$$$^p < 0.0001 pairwise comparisons between the groups. ARDS: Acute respiratory distress syndrome; DEX: Dexamethasone; GalN: Galactosamine; LPS: Lipopolysaccharide; NS: Normal saline; RJX: Rejuveinix; SOD: Superoxide dismutase.

**Figure 3.**
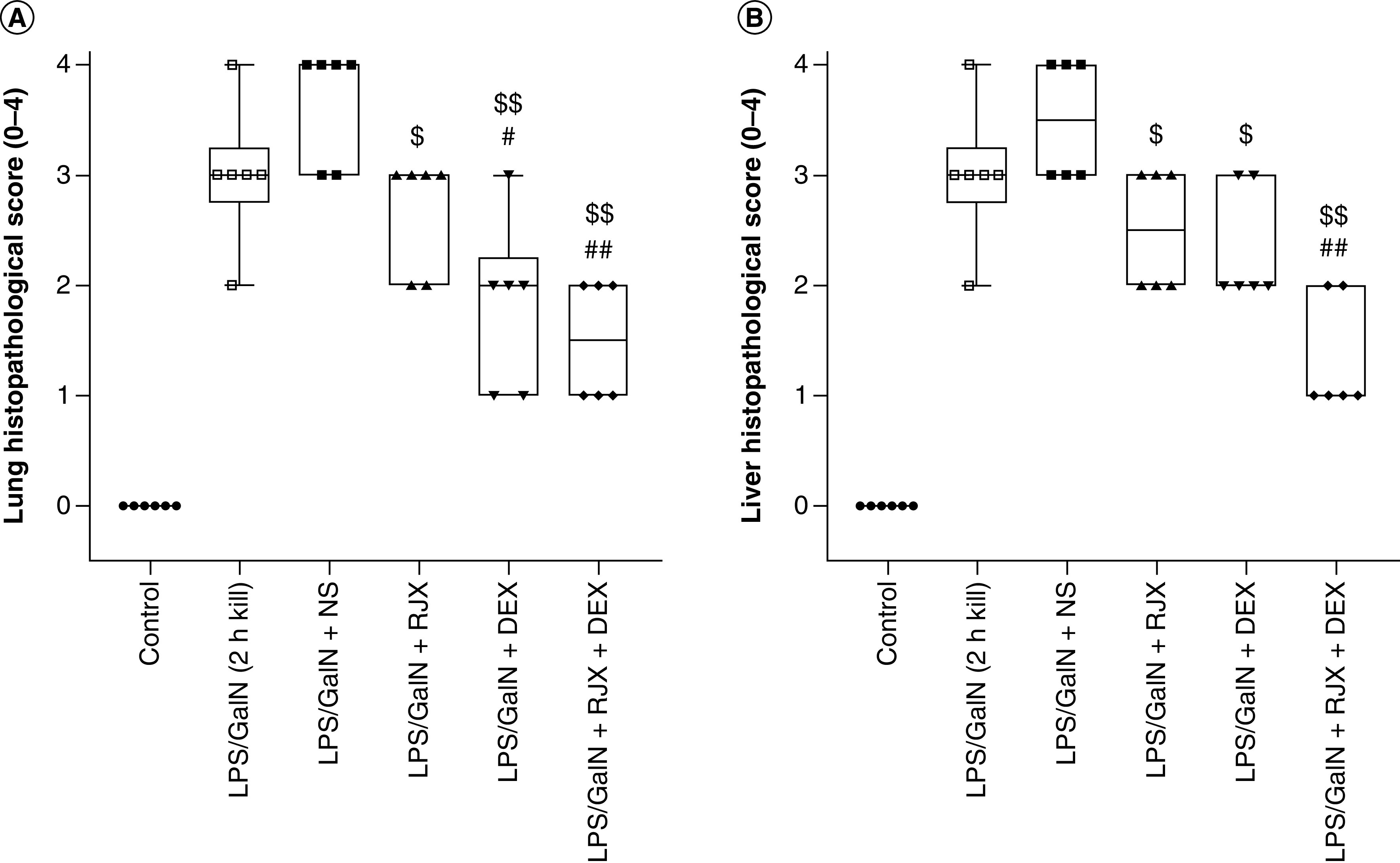
*In vivo* treatment activity of low-dose RJX, supratherapeutic high-dose DEX and their combination on lung and liver histopathological scores in the LPS-GalN mouse model of fatal cytokine storm and sepsis. We used previously published methods and statistical analysis tools [17,21]. Groups of six BALB/c mice were treated with intraperitoneal injections of RJX (sixfold diluted, 4.2 ml/kg, 0.5 ml/mouse), DEX (6.0 mg/kg, 0.5 ml/mouse) or vehicle (NS, 0.5 ml/mouse) 2 h post-injection of LPS-GalN. Except for untreated control mice (control), each mouse received 0.5 ml of LPS-GalN (consisting of 100 ng of LPS plus 8 mg of D-galactosamine) intraperitoneally. The tissue damage in the **(A)** lungs and **(B)** liver were scored using previously published grading scales [17,21]. Mann–Whitney *U* test was used for comparing the results among different treatment groups. Statistical significance between groups is shown by ^#^p < 0.05; ^##^p < 0.01 as compared with LPS/GaIN (2 h kill) group, ^$^p < 0.05; ^$$^p < 0.01 as compared with LPS/GaIN + NS group. DEX: Dexamethasone; GalN: Galactosamine; LPS: Lipopolysaccharide; NS: Normal saline; RJX: Rejuveinix.

As there was significant residual tissue damage in the lungs and liver of surviving LPS-GalN-challenged mice treated with RJX or DEX (even at the 6.0 mg/kg high dose level), we next sought to determine if a combination of low-dose RJX (0.7 ml/kg) and high-dose DEX (6.0 mg/kg) could improve the tissue healing and the survival outcome after LPS-GalN exposure [23]. Treatments were initiated at 2 h after LPS-GalN injection at a time of documented active systemic inflammation. LPS-GalN caused severe inflammation and oxidative stress in the lungs and liver with enhanced lipid peroxidation, as measured by a marked elevation of malondialdehyde levels, and decreased tissue levels of vitamin C as well as the antioxidant enzymes GSH-Px that inhibit lipid peroxidation and SOD that converts superoxide anion radicals that contribute to lipid peroxidation into hydrogen peroxide and oxygen. Low-dose RJX alone or in combination with DEX significantly suppressed the oxidative stress, as documented by increases in vitamin C, GSH-Px and SOD levels that were reduced by LPS-GalN (Figure 2A, C & E depict the liver tissue levels; B, D & F depict the lung tissue levels). Notably, delayed treatments with RJX, DEX or RJX + DEX starting at 2 h after the LPS-GalN injection partially reversed the inflammatory lung damage within 24 h [23], as evidenced by significantly reduced histopathological lung/ALI scores (Figures 3 & Supplementary Figure 6). The tissue healing activity of the combination was more pronounced than the tissue healing activity of RJX alone or DEX alone and resulted in a near-complete recovery of the severe lung damage (Figures 3 & Supplementary Figure 6).

RJX + DEX combination also decreased the liver tissue damage (Figure 3) [23]. While 12 of 12 mice (100%) treated with either RJX alone (n = 6) or DEX alone (n = 6) had moderate-to-severe residual damage in either their lungs or liver, two of the six mice (33.3%) treated with the combination regimen had no or minimal damage (i.e., histopathological damage scores: 0–1) in both organs (p = 0.098, Fisher's exact test). RXX + DEX was more effective than RJX alone or DEX alone in improving the survival outcome (Supplementary Figure 7). In contrast to the rapid death of all control mice treated with NS (median survival: 4.3 h post-LPS-GalN or 2.3 h after administration of NS), 100% of mice treated with RJX + DEX survived the LPS-GalN challenge (median survival: >24 h post LPS-GalN or >22 h after initial administration of RJX + DEX) (Supplementary Figure 7). By comparison, the combined group of mice treated with monotherapy (i.e., RJX alone or DEX alone) (n = 12) had a 24-h survival rate of 41.7% (monotherapy with RJX or DEX vs combination therapy with RJX + DEX: log-rank X^2^ = 3.053; p = 0.081).

**Figure 4.**
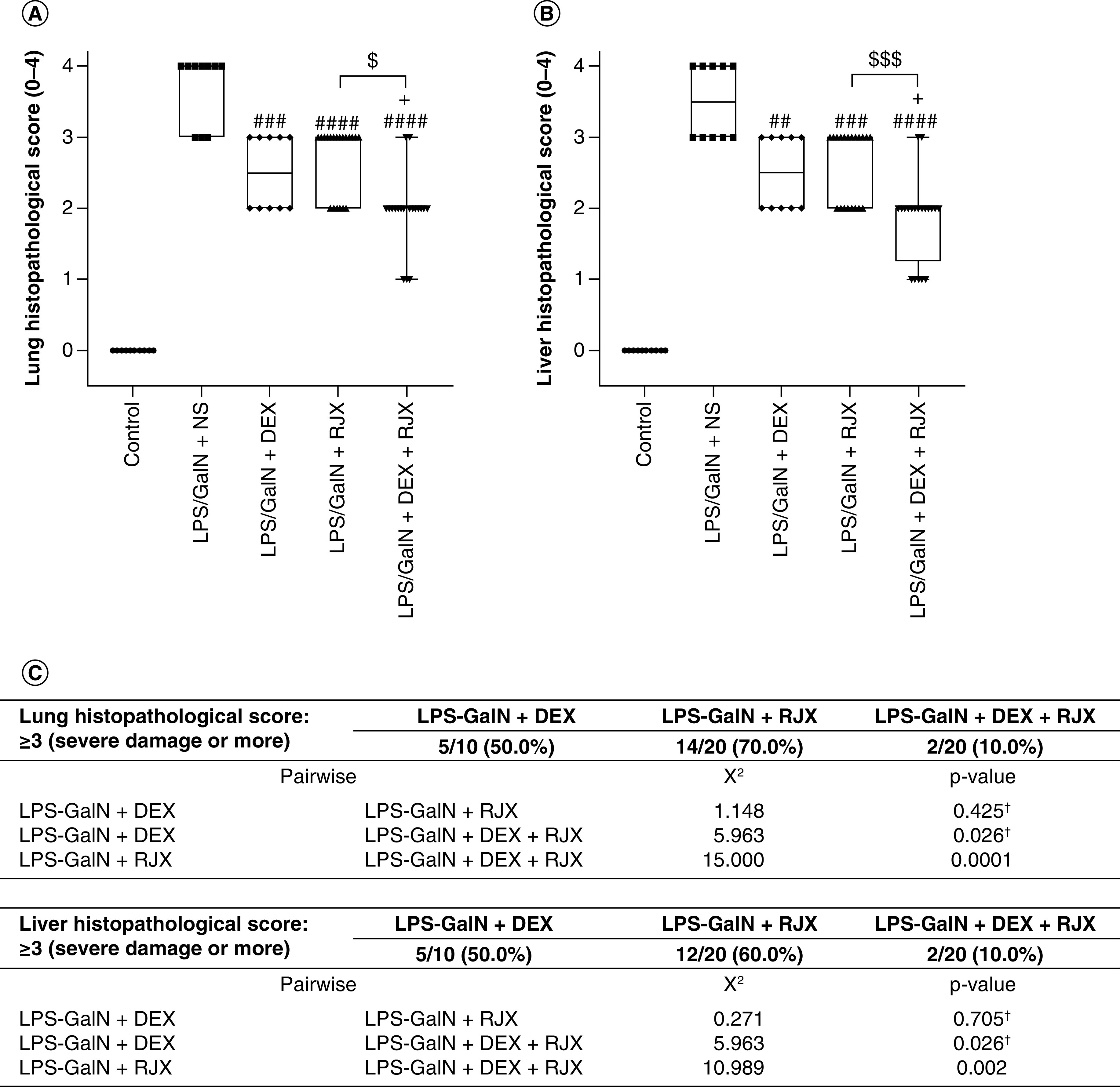
*In vivo* treatment activity of RJX, DEX and RJX + DEX on lung and liver histopathological scores in the LPS-GalN mouse model of fatal cytokine storm, sepsis, systemic inflammation, ARDS and multiorgan failure. BALB/c mice were treated with intraperitoneal injections of RJX (n = 20, sixfold diluted, 4.2 ml/kg, 0.5 ml/mouse), DEX (n = 10, 0.6 mg/kg, 0.5 ml/mouse), RJX + DEX (n = 20, 0.5 ml/mouse) or vehicle (NS, 0.5 ml/mouse) 2 h post-injection of LPS-GalN. Except for untreated control mice (control, n = 10), each mouse received 0.5 ml of LPS-GalN (consisting of 100 ng of LPS plus 8 mg of D-galactosamine) intraperitoneally. The depicted whisker plots represent the median and values. The tissue damage in the **(A)** lungs and **(B)** liver were scored using previously published grading scales [17,21]. Kruskal–Wallis test and Mann–Whitney *U* test were used for comparing the results among different treatment groups. **(C)** For severe damage, lung and liver histological scores were compared by Pearson's Chi-Square or Fisher's Exact test. ^†^Fisher's exact Chi-Square test used. Statistical significance between groups is shown by ^##^p < 0.01; ^###^p < 0.001; ^####^p < 0.0001 as compared with LPS/GaIN + NS group; ^+^p < 0.05 as compared with LPS/GaIN + DEX group and ^$^p < 0.05; ^$$$^p < 0.001 pairwise comparisons between the groups. ARDS: Acute respiratory distress syndrome; DEX: Dexamethasone; RJX: Rejuveinix.

**Figure 5.**
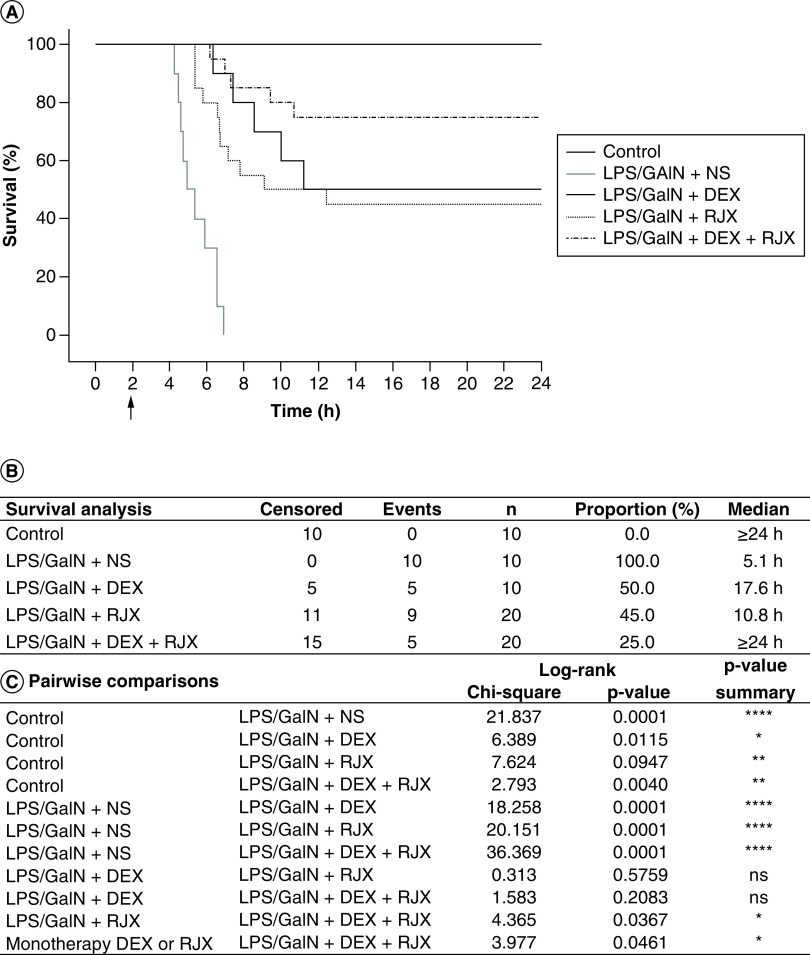
*In vivo* treatment activity of low-dose RJX, standard-dose DEX and their combination in the LPS-GalN mouse model of fatal cytokine storm, sepsis, systemic inflammation, ARDS and multiorgan failure. BALB/c mice were treated with intraperitoneal injections of RJX (n = 20, sixfold diluted, 4.2 ml/kg, 0.5 ml/mouse), DEX (n = 10, 0.6 mg/kg, 0.5 ml/mouse), RJX + DEX (n = 20, 0.5 ml/mouse) or vehicle (NS, 0.5 ml/mouse) 2 h post-injection of LPS-GalN. Except for untreated control mice (control, n = 10), each mouse received 0.5 ml of LPS-GalN (consisting of 100 ng of LPS plus 8 mg of D-galactosamine) intraperitoneally. The cumulative proportion of mice remaining alive (survival, %) is shown as a function of time after the LPS-GalN challenge. Depicted are the Kaplan–Meier survival curves **(A)** and survival data with statistical analysis **(B & C)** of the different treatment groups. ARDS: Acute respiratory distress syndrome; DEX: Dexamethasone; LPS: Lipopolysaccharide; NS: Normal saline; RJX: Rejuveinix.

We next evaluated the efficacy of a combination regimen that employs low-dose RJX (0.7 ml/kg) plus standard-dose DEX (0.6 mg/kg) [23]. Notably, delayed treatments with low-dose RJX plus standard-dose DEX starting at 2 h after the LPS-GalN injection reversed the lung damage, as evidenced by significantly reduced histopathological lung scores (Figure 4). The tissue-healing activity of the combination was more pronounced than the tissue-healing activity of RJX alone or DEX alone (Figure 4A). Hence, although treatments were delayed until the onset of fulminant cytokine storm and systemic inflammation as well as very severe lung damage, a near-complete recovery of the inflammatory lung injury was achieved in the vast majority of mice within 24 h. Similar to its effects on the LPS-GalN induced ALI, RJX + DEX combination significantly reduced the liver injury (Figure 4B). While five of ten mice (50%) treated with standard-dose DEX alone and 14 of 20 mice (70%) treated with low-dose RJX alone had severe residual lung injury (histopathological lung score ≥3), only two of 20 (10%) of mice treated with the DEX + RJX combination had severe residual lung damage. The observed superiority of the combination regimen was statistically significant (Figure 4C). Similarly, while five of ten DEX-treated mice (50%) and 12 of 20 RJX-treated mice (60%) had severe residual liver damage, only two of 20 mice (10%) treated with the DEX + RJX combination had severe residual liver damage, and this difference was statistically significant (Figure 4C). In contrast to the rapid death of all control mice treated with NS (median survival: 5.1 h post LPS-GalN or 3.1 h after the administration of NS), 15 of 20 (75%) of mice treated with RJX + DEX survived the LPS-GalN challenge (median survival: >24 h post LPS-GalN) (Figure 5). By comparison, the combined group of mice treated with monotherapy (i.e., RJX alone or DEX alone) (n = 30) had a 24-h survival rate of 53% (i.e., 16 of 30 mice) (monotherapy with RJX or DEX vs combination therapy with RJX + DEX: log-rank X^2^ = 3.977; p = 0.046).

### Clinical data from the phase I study of RJX in high-risk COVID-19 patients

Thirteen hospitalized adult patients with high-risk COVID-19 received daily intravenous infusions of RJX (maximum one cycle = 7 consecutive days) in combination with institutional SOC. Preliminary data from this phase I study indicated that RJX plus DEX and other components of the SOC were well tolerated. The results are detailed in Supplementary Figures 8–16 & Supplementary Tables 1–6. Improvements have been observed in serum inflammatory markers as well as clinical parameters. The outcome data for 12 evaluable patients are shown in Supplementary Table 6. This early data must be interpreted with due caution within the confines of a nonrandomized phase I study.

## Discussion

Sepsis is a major cause of mortality [1,3] and new treatment regimens are urgently needed for sepsis patients. COVID-19 has become a leading cause of death globally [29–31]. Patients with high-risk COVID-19 with viral sepsis are in urgent need of effective strategies that can prevent and/or reverse the systemic inflammatory process and its often fatal complications, including acute respiratory distress syndrome (ARDS) and multiorgan failure [11–13,15–17,29–31]. DEX has been shown to improve the survival outcome of patients with ARDS [32]. Similar findings have been reported from other studies [33,34].

RJX is an emerging anti-inflammatory drug candidate to prevent and treat sepsis, including bacterial sepsis in neutropenic cancer patients as well as viral sepsis in COVID-19 patients [17]. The active ingredients of RJX have demonstrated potential as antisepsis therapeutics [17]. However, while several studies appeared to demonstrate therapeutic benefit, no meaningful clinical activity was observed in other studies [20,22,24–27,35–37]. Additional studies are needed to clarify the reported discrepancies, which may in part relate to differences in eligibility criteria and timing of interventions.

Inflammatory cytokines play an important and causal role in the development of potentially life-threatening complications of COVID-19-associated viral sepsis in adults as well as multisystem inflammatory syndrome in children [9,38–46]. Recently, we demonstrated that RJX prevents in the LPS-GalN mouse model of sepsis the marked increase of each of these cytokines in the serum as well as lungs and liver [17]. Furthermore, preliminary evidence has suggested that it may also be effective in reversing sepsis if administered after the onset of sepsis [19]. Here we report on the activity of a combination of RJX plus DEX in therapeutic settings in a preclinical sepsis model. The combination of RJX plus DEX immediately and profoundly decreased the inflammatory cytokine responses to LPS-GalN, mitigated the inflammatory tissue damage in the lungs and liver, and prevented a fatal outcome. Although the treatments were started after the onset of fulminant cytokine storm characterized by markedly elevated serum cytokine levels and systemic inflammation as well as severe lung damage, a near-complete recovery of the inflammatory lung injury was achieved within 24 h, and the survival outcome was improved. The superiority of the combination regimen was particularly striking when we compared the histopathological data on sepsis-associated organ damage in lungs and liver from mice treated with monotherapy (RJX or DEX alone) versus combination therapy. Notably, initiation of combination therapy after onset of the inflammatory cytokine responses and systemic inflammation resulted within 24 h in a near-complete recovery of very severe organ damage in the lungs that was caused by LPS-GalN-induced sepsis.

Glucocorticoids, including DEX, have multiple effects on the immune system and exhibit potent anti-inflammatory activity [47]. DEX has been shown to improve the survival outcome of patients with ARDS [34,48–52]. Furthermore, recent clinical studies, including the open-label randomized RECOVERY trial, have demonstrated that the standard anti-inflammatory drug DEX improves the survival outcome of hospitalized high-risk COVID-19 patients [49,50].

Our preliminary data from the clinical phase I study showed that the majority of high-risk COVID-19 patients had a rapid clinical recovery with resolution of the hyperinflammatory response and oxygen therapy requirements. Additional studies in a randomized setting are needed to confirm the reported preliminary findings and assess any potential benefit from the addition of RJX to the SOC that includes DEX. RJX will be evaluated in a randomized, placebo-controlled study in hospitalized COVID-19 patients with viral sepsis to test the hypothesis that it will contribute to a faster resolution of respiratory failure and a reduced case mortality rate when combined with DEX and other components of the SOC. A randomized phase II proof-of-concept study has also been designed to determine if RJX plus DEX combination can reduce the mortality rate of high-risk bacterial sepsis.

## Conclusion

The combination of RJX plus DEX immediately and profoundly decreased the inflammatory cytokine (IL-6, TNF-α) responses to LPS-GalN, mitigated the inflammatory tissue damage in the lungs and liver, and prevented a fatal outcome. Although the treatments were started after the onset of fulminant cytokine storm characterized by markedly elevated serum IL-6 and TNF-α levels and systemic inflammation as well as severe lung damage, a near-complete recovery of the inflammatory lung injury was achieved within 24 h, and the survival outcome was improved. Our preliminary data from the clinical phase I study showed that the majority of high-risk COVID-19 patients treated with RJX in combination with DEX had a rapid clinical recovery with resolution of the hyperinflammatory response and oxygen therapy requirements. Additional studies in a randomized setting are needed to confirm the reported preliminary findings and assess any potential benefit from the addition of RJX to the SOC that includes DEX.

**Summary points**

- The primary objective of the present study was to compare the effects of Rejuveinix (RJX), the standard anti-inflammatory drug dexamethasone (DEX) and their combination on the severity of sepsis and survival outcome in an animal model that employs LPS plus D-galactosamine (LPS-GalN) to induce fatal sepsis.
- We hypothesized that RJX, especially when combined with DEX, would improve the survival outcome of mice who develop systemic inflammation following injection with a lethal dose of LPS-GalN.
- We demonstrate that RJX – at a dose level >ten-times lower than its clinical maximum tolerated dose – exhibits potent single-agent anti-inflammatory activity and is as effective as DEX at standard dose levels in this model of fatal sepsis.
- The combination of RJX plus DEX immediately and profoundly decreased the inflammatory cytokine (IL-6, TNF-α) responses to LPS plus D-galactosamine, mitigated the inflammatory tissue damage in the lungs and liver, and prevented a fatal outcome.
- Although the treatments were started after the onset of fulminant cytokine storm characterized by markedly elevated serum IL-6 and TNF-α levels and systemic inflammation as well as severe lung damage, a near-complete recovery of the inflammatory lung injury was achieved within 24 h, and the survival outcome was improved.
- Preliminary data from a clinical phase I study indicated that RJX plus DEX and other components of the standard of care were well tolerated.
