# Supplementary material for: Evaluation of the potential of Rejuveinix plus dexamethasone against sepsis
Source: Future Microbiol. 2022 Sep 2:10.2217/fmb-2022-0044. doi: 10.2217/fmb-2022-0044 (PMC9443789; doi:10.2217/fmb-2022-0044)
Supplement: Supplementary file 1 [file supplementary_material.zip › Supplementary_Information_REVISED_JULY_9.docx]

**SUPPLEMENTAL INFORMATION**

**Evaluation of the potential of RJX plus dexamethasone against sepsis**

**Study Design and Eligibility Criteria for Clinical Phase 1 Study**

The safety, tolerability, and feasibility of using RJX in combination with clinical standard of care for hospitalized COVID-19 patients were evaluated in an open-label pilot study that was Part 1 of an ongoing double-blind, randomized, Phase 1/2 Study (ClinicalTrials.gov Identifier: NCT04708340). 13 patients who were hospitalized with COVID-19 pneumonia and abnormally elevated serum inflammatory biomarkers markers were with IV RJX (daily x 7 days) plus SOC. The primary goal of the study was to evaluate the safety and tolerability of RJX plus SOC in hospitalized severe COVID-19 patients. The secondary goal was to evaluate the clinical efficacy of RJX plus SOC in hospitalized severe COVID-19 patients. The Eligibility Criteria are detailed in the clinical protocol synopsis posted at ClinicalTrials.gov ((ClinicalTrials.gov Identifier: NCT04708340). Patients received daily 40 min (±10 min) infusions of 20 mL RJX mixed with 100 mL normal saline (total volume = 120 mL) intravenously plus SOC for 7 consecutive days. Some patients who no longer required inpatient care due to rapid improvement of their medical condition were discharged before day 7 and consequently received fewer than 7 treatments of RJX. SOC was provided until the patient was discharged. AEs were graded according to National Cancer Institute (NCI) Common Terminology Criteria for Adverse Events Version 5.0 (NCI CTCAE (v5.0). An 8-point ordinal scale was used as an assessment of the clinical status at the first assessment of a given study day).

**Eligibility Criteria in Pilot Clinical Study**

The Inclusion Criteria were:

**Cohort 1:**

1. Hospitalized COVID-19 patients ≥18 years without hypoxemia who are either not receiving any oxygen therapy OR are receiving supplemental oxygen via mask or nasal prongs (namely, clinical status score 4 or 5 on an 8-point ordinal scale)
2. Hospitalized COVID-19 patients age ≥65 years AND type 2 diabetes or hypertension, OR
3. Hospitalized COVID-19 patients ≥18 years AND abnormal blood tests with CRP >50 mg/L PLUS at least 1 of the following biomarkers:
   1. D-dimer >1,000 ng/mL
   2. Ferritin >500 µg/L
   3. High sensitivity cardiac troponin >2 × ULN
   4. LDH >245 U/L

**Cohort 2**

1. Hospitalized COVID-19 patients with hypoxemia who are either receiving NIPPV OR high-flow oxygen (namely, clinical status score 3 on an 8-point ordinal scale).
2. Bilateral opacities on a chest x-ray OR chest CT scan.

**Cohort 1 and Cohort 2**

1. Male and non-pregnant, non-lactating female patients with SARS-CoV-2 infection that is documented by a Food and Drug Administration (FDA)-authorized diagnostic reverse transcription polymerase chain reaction (RT-PCR) or antigen test at/or within 4 days of Screening
2. ≥18 years of age
3. Body weight ≥40 kg at Screening
4. History of COVID-19 within the last 2 weeks prior to study enrollment
5. The patient OR a legally authorized representative has provided written informed consent
6. Females of childbearing potential must have a negative beta‑human chorionic gonadotropin pregnancy test at Screening
7. Females of childbearing potential must agree to be abstinent or else use a medically acceptable form of contraception from the Screening period through Day 28. Medically acceptable forms of contraception including implants, injectables, combined oral contraceptives, some intrauterine devices, sexual abstinence or vasectomy, and double-barrier method [condom and occlusive cap (diaphragm or cervical/vault caps)] with spermicidal foam/gel/film/suppository
8. Code status documented in the medical chart/record – DNR/DNI code status is an exclusion criterion for this study

The Exclusion Criteria were:

**Cohort 1**

1. Receiving high-flow oxygen OR NIPPV.
2. ARDS by Berlin definition

**Cohort 2**

1. Moderate ARDS by Berlin definition. Patients who are considered as having mild ARDS are eligible if they are not intubated.

Patients considered to have moderate or severe ARDS are not eligible for RPI-015 Cohort 1 or Cohort 2.

**Cohort 1 and Cohort 2**

1. On extracorporeal membrane oxygenation
2. Uncontrolled hypertension (systolic blood pressure [BP] >150 mmHg and/or diastolic BP >100 mmHg), unstable angina, congestive heart failure of New York Heart Association Classification Class III or IV (i.e., Class III: marked limitation in activity due to symptoms, even during less-than-ordinary activity, e.g., walking short distances [20‑100 m], comfortable only at rest; Class IV: severe limitations, experiences symptoms even while at rest, mostly bedbound patients), serious cardiac arrhythmia requiring treatment (exceptions: atrial fibrillation, paroxysmal supraventricular tachycardia), history of myocardial infarction within 12 months prior to enrollment
3. Subjects with a history of congenital long QT syndrome or of Torsades de pointes; subjects with bradycardia (<60 bpm), heart block (excluding 1^st^ degree block, being PR interval prolongation only); subjects with any of the following findings on electrocardiogram (ECG): QTc interval >470 msec in women OR >450 msec in men; subjects requiring any drugs known to prolong the QTc interval, including antiarrhythmic medications

**Clarification:** If a patient is meeting all other eligibility criteria, has an otherwise normal ECG, and the PI considers the patient a good candidate for the study and has no medical concerns regarding the patient’s enrollment in the study, a waiver may be provided by the sponsor from QTc requirements for 450 msec < QTc < 480 msec (men) or 470 msec < QTc <500 msec (women).

If a waiver is provided, follow-up ECGs should be obtained as per Section 1.8 of the protocol and if patient's QTc shows further prolongation, a cardiology consultation should be obtained.

Subjects requiring medications that prolong QTc intervals are excluded. Exception: Patients whose standard of care for COVID-19 involves such drugs and whose QTc are within normal range.

1. Shock or hypotension requiring vasoactive amines, such as dopamine, norepinephrine, epinephrine, or dobutamine
2. Renal function impairment with Serum creatinine ≥2 mg/dL or Estimated glomerular filtration rate <50 mL/min/1.73 m^2^
3. Liver function impairment with Total bilirubin ≥2 mg/dL or ≥1.5xULN
4. Platelet count <50,000/µL
5. Multi-organ failure
6. History of an allergic reaction or hypersensitivity to the study drug or any component of the study drug formulation
7. Use of systemic corticosteroids, nonsteroidal anti-inflammatory drugs, antibiotics, and antiviral drugs that are not part of the standard of care
8. Presence of any uncontrolled concomitant illness (e.g., bacterial sepsis or invasive fungal infection), or other serious illness and medical conditions, or other medical history, including laboratory results, which, in the Investigator’s opinion, would be likely to interfere with their participation in the study
9. Pregnancy or breast-feeding (for women)
10. Do not resuscitate (DNR)/Do not intubate (DNI) Code status chose by subject or LAR

**Patients and Patient Disposition.**

21 patients were screened. There were 8 screen failures. 6 patients were enrolled in Cohort 1 and 7 patients were enrolled in Cohort 2. One of the Cohort 2 patients withdrew consent on day 4. Consequently, 13 patients were evaluable for safety evaluation and 12 patients were evaluable for efficacy evaluation.

**Patient Evaluations**

An 8-point ordinal scale was used as an assessment of the clinical status at the first assessment

of a given study day

***8-point Ordinal Scale:***

1. Death

2. Hospitalized, on invasive mechanical ventilation or ECMO

3. Hospitalized, on non-invasive ventilation or high flow oxygen devices

4. Hospitalized, requiring supplemental oxygen

5. Hospitalized, not requiring supplemental oxygen - requiring ongoing medical care (COVID-19 related or otherwise)

6. Hospitalized, not requiring supplemental oxygen - no longer requires ongoing medical care

7. Not hospitalized, limitation on activities and/or requiring home oxygen

8. Not hospitalized, no limitations on activities

The performance status during follow-up evaluations was examined using the Eastern Cooperative Group (**ECOG**) scale:

0 - Fully active, able to carry on all pre-disease performance without restriction.

1 - Restricted in physically strenuous activity but ambulatory and able to carry out work of a light or sedentary nature, e.g., light house work, office work.

2 - Ambulatory and capable of all selfcare but unable to carry out any work activities. Up and about more than 50% of waking hours.

3 - Capable of only limited selfcare, confined to bed or chair more than 50% of waking hours.

4 - Completely disabled. Cannot carry on any selfcare. Totally confined to bed or chair.

5 - Dead.

**Phase 1 Study Conduct**

The study was performed under IND149585 at the following 4 centers in the US as an open-label study sponsored by Reven Pharmaceuticals: (1) Memorial Hermann Memorial City Medical Center, Houston, Texas; (2) Christus Health Santa Rosa Hospital, New Braunfels, Texas; (3) LFROS Research and United Memorial Medical Center, Houston, Texas; (4) PRX Research and Dallas Regional Medical Center, Dallas, Texas.

**Patient Disposition and Patient Characteristics**

Twenty-one patients were screened for the clinical study. There were 8 screen failures. Thirteen hospitalized adult patients with severe or critical COVID-19, including 8 males and 5 females with a median age of 46 years (Range: 24-72 years) of whom 11 were White (Hispanic or Latino) and 2 were Asian (not Hispanic or Latino) were enrolled in one of the 2 cohorts of the clinical study within 0-11 days (Median: 4 days; Mean ± SE=4.5±1.0) days after initial COVID-19 diagnosis (**Table S1**). All 13 patients had imaging evidence of viral pneumonia and they were hypoxic requiring oxygen therapy. All patients were symptomatic with cough and shortness of breath. Nine patients had a fever as well. The mean and median BMI values were 35.6±1.9 and 35.1, respectively (Range: 23.8-49.4). Ten patients were obese with BMI values ranging from 32.1 to 49.4 and 2 patients were overweight with BMI values of 28.3 and 29.3, respectively. Five patients had HTN, 3 patients had diabetes, and 3 patients had pulmonary co-morbidities (asthma or COPD). Only one patient was vaccinated. All 13 patients had elevated serum CRP values ranging from 42.4 to 297.6 (Median: 64.3; Mean ± SE = 91.8±19.1). The ALC values ranged from 0.3 to 1.4 (Median: 0.9; Mean ± SE= 0.8±0.1). Eleven patients had lymphocytopenia with ALC <1,000/µL. All 6 patients treated in Cohort 1 had severe COVID-19 with Chest X-ray/CT-documented pneumonia associated with hypoxemia and respiratory distress, elevated baseline levels of inflammation markers in blood, such as abnormally high levels of CRP, IL-6, Ferritin, and/or LDH, and had a baseline score of 4 based on the 8-point ordinal scale (**Table S1**). By comparison, all 7 patients treated in Cohort 2 had critical COVID-19 with Chest X-ray/CT-documented pneumonia associated with hypoxemic respiratory failure requiring high flow oxygen therapy and/or non-invasive positive pressure ventilation (NIPPV), increased inflammation markers and had a baseline score of 3 based on the 8-point ordinal scale (**Table S1**). One Cohort 2 patient withdrew consent on day 4 and was replaced with a new patient to have a total of 6 patients evaluable for response.

**Ethics Statement and Study Approval**

The study was performed according to the guidelines of the International Conference on Harmonization (ICH) for Good Clinical Practice (ICHE6/GCP). A written informed consent was obtained from patients prior to enrollment. The study protocol was approved by the WCG-Central Institutional Review Board (IRB) (OHRP/FDA Parent Organization number: IORG0000432; OHRP/FDA IRB registration number: IRB00000533). The Central IRB-approved study/protocol number was RPI015 (IRB Tracking Number: 20203418).

**Statistical Analyses of Clinical Data**

Standard statistical methods were applied for the analysis of the clinical data.  Descriptive statics for patient cohorts were calculated using the R platform (R version 4.1.2 (2021-11-01)) ran in Rstudio (2021.09.0 Build 351) using standard functions provided by the statistical package “plyr_1.8.6”.  Swimmer plots to visualize individual patient treatment outcomes and waterfall plots to visualize grouped and ranked outcomes were constructed using graph drawing packages implemented in the R programming environment: swimplot_1.2.0, colorspace_2.0-2, ggplot2_3.3.5, ggbreak_0.0.7, cowplot_1.1.1, ggh4x_0.2.1 and ggthemes_4.2.4.  The Kaplan–Meier method, log-rank chi-square test, was used to investigate survival of patients utilizing software packages survival_3.2-13, survminer_0.4.9 and survMisc_0.5.5 in R environment.  The kinetics of the CRS-related serum biomarkers were investigated using first order exponential “decay” functions of percentage of each biomarker.  The combined results for each biomarker were graphed on semi-log axis (Log_10_ (% inhibition) = slope x Time + Intercept).  The time to 50% reduction of the serum concentration of each biomarker was calculated from the intercept and the slope of the decay function ((Log_10_ (50) – Intercept)/slope).  These operations were carried out in the R programming environment: plyr_1.8.6 and ggplot2_3.3.5.

**Clinical Study Data**

*Safety.* Patient characteristics and demographic features are shown in **Table S1**. All patients were treated daily with IV RJX plus institutional SOC (**Table S2**). One patient withdrew consent on day 4 without any treatment-emergent AEs. **Table S3** details all Grade 3-5 AEs reported. No patient developed a DLT or SAE while still receiving RJX. None of the reported Grade 3-5 AEs was deemed related to RJX – they were all reported as related to COVID-19. Listing of all grade SAEs is provided in **Table S4**. **Table S5** depicts the incidence of Grade 3-5 AEs and SAEs by MedDRA PT and listing of deaths for all enrolled patients.

*Pharmacodynamics*. Protocol therapy resulted in a rapid resolution of pneumonia and systemic inflammation in 9 of 12 patients enrolled. In these 9 patients, we observed a rapid normalization in the levels of the inflammation biomarkers CRP, Ferritin, LDH, and IL-6. We investigated the first order kinetics of the CRP decline in each of the 9 responding patients by fitting a straight line to a semi-log plot of the portion of the biomarker curve over the course of RJX treatment when a maximum reduction in values was observed. The slope of the line represents the rate constant for CRP reduction in log_10_ scale, and times to 50% reduction of CRP values were calculated using the rate constant (**Figure S8**). Serum CRP levels showed a rapid decline with an average (Mean±SE) time of 1.6±0.2 days to reach 50% of baseline values (Median: 1.7 days, Range: 0.9-2.5 days) (**Figure S9**). Similar results were observed for IL-6 (N=5; Mean: 13.1 ± 6.1 days, Median: 6.3 days, Range: 1.6 - 29.3 days (**Figure S10**), Ferritin (N=8; Mean: 8.8 ± 3.4 days, Median: 5.2 days, Range: 2.9 - 32.1 days) (**Figure S11**) and LDH (N=5; Mean: 8.6 ± 1.8 days, Median: 7.2 days, Range: 4.9 - 14.6 days) levels (**Figure S12**). By comparison, the kinetics of normalization of elevated TGF-b levels was much slower and near-normal levels were achieved in some patients only after 1-2 months (N=4; Mean±SE: 26.6±17.3 days; Median 13.2 days; Range: 2.3-77.6 days) (**Figure S13**).

*Efficacy*. The outcome data for 12 evaluable patients are shown in **Table S6** (6 from Cohort 1 and 6 from Cohort 2; Patient 008-1204 who withdrew his consent on day 4 is not shown in this table). Nine (9) of the 12 patients, including each of the 6 Cohort 1 patients and 3 of 6 Cohort 2 patients with hypoxemic respiratory failure, showed rapid clinical recovery with normalization of the blood oxygen levels. The overall survival curve is shown in **Figure S14**; the median survival was not reached. **Figure S15** shows the kinetics of improvement or worsening of the clinical health status scores according to the 8-point ordinal scale for each patient. The score changes were charted utilizing Swimmer plots. **Figure S16** shows a waterfall plot depicting the maximum increase (improvement) or decrease (worsening) of the 8-point Ordinal Scale scores. The score change ranged from -2 to +5 (Median: +3.5; Mean ± SE: 2.4 ± 0.8). The score change for Cohort 1 patients (N=6) ranged from +3 to +4 (Median: +3.5, Mean ± SE : 3.5± 0.2). By comparison, the score change for Cohort 2 patients (N=6) ranged from -2 to +5 (Median: +1; Mean ± SE: 1.3±1.5). Each of the 6 patients in Cohort 1 had a baseline score of 4. Their oxygen supplementation requirements have resolved, and they were consequently discharged from the hospital between 3 to 7 days (median: 4.5 days) after initiation of protocol therapy. Hence, their scores showed a +3 (Score 7: Not hospitalized, limitation on activities and/or requiring home oxygen) to +4 improvement within a week (Score 8: Not hospitalized, no limitations on activities – corresponds to ECOG score of 0). Of the 6 patients with hypoxemic respiratory failure who had a baseline score of 3 and required high flow oxygen and/or NIPPV, 3 were discharged from hospital at 7,7, and 14 days respectively upon resolution of their hypoxemic respiratory failure with a +4-+5 improvement on the 8-point ordinal scale (**Figure S15**). Two of the remaining 3 patients experienced progression of their COVID-19 requiring intubation on day 8 and one patient developed COVID-19 related complications, including sepsis with coagulopathy (day 10-17), pulmonary embolism (day 19), and fatal mesenteric ischemia (day 23) (**Figure S15, Table S5**).

**Supplemental Figure Legends**

**Figure S1.** The effects of Rejuveinix (RJX) and the different doses of the Dexamethasone (DEX), treatments on serum interleukin 6 (IL-6; Panel A), tumor necrosis factor-alpha (TNF-α; Panel B) in a Mouse Model of Fatal Cytokine Storm, Sepsis, Systemic Inflammation, ARDS and Multiorgan Failure. BALB/C mice were treated with i.p injections of RJX (0.7 ml/kg = 4.2 ml/kg of 6-fold diluted RJX, 0.5 ml/mouse; or 1.4 ml/kg = 8.4 ml/kg of 1:6-diluted RJX, 0.5 ml/mouse), DEX (0.1 mg/kg, 0.6 mg/kg and 6.0 mg/kg), or vehicle (NS, 0.5 mL/mouse) two hours post-injection of LPS-GalN, or terminated at 2 hours post LPS-GalN injection without any therapeutic intervention. Except for untreated control mice (Control), each mouse received 0.5 ml of LPS-GalN (consisting of 100 ng of LPS plus 8 mg of D-galactosamine i.p.). The depicted Whisker plots represent the median and values for serum IL-6 and TNF-α levels from all 6 mice from each group except for the 1.4 ml/kg RJX group where blood samples were obtained from all 10 mice. In Panel A, Welch's ANOVA and Tamhane’s T2 post-hoc test were used for comparing the results among different treatment groups. In Panel B, ANOVA and Tukey post-hoc test were used for comparing the results among different treatment groups. Statistical significance between groups is shown by **** p<0.0001 as compared to control group, and ## p<0.01; ### p<0.001; #### p<0.0001 as compared to LPS/GalN (2h kill) group, ++ p<0.01; +++ p<0.001; ++++ p<0.0001 as compared to LPS/GalN+NS group, $ p<0.05; $$ p<0.01; $$$ p<0.001; $$$$ p<0.0001 LPS/GalN+RJX (4.2) group, && p<0.01; &&& p<0.001; &&&& p<0.0001 as compared to LPS/GalN+RJX (8.4) group, and and δδ p<0.01; δδδδ p<0.0001 pairwise comparison.

**Figure S2.** Tissue-Level In Vivo Activity of Rejuveinix (RJX) and the different doses of the Dexamethasone (DEX), treatments on Lung and Liver Histopathological Scores in a Mouse Model of Fatal Cytokine Storm, Sepsis, Systemic Inflammation, ARDS and Multiorgan Failure. BALB/C mice were treated with i.p injections of RJX (6-fold diluted, 4.2 mL/kg or 8.4 mL/kg, 0.5 ml/mouse), DEX (0.1 mg/kg, 0.6 mg/kg and 6.0 mg/kg), or vehicle (NS, 0.5 mL/mouse) two hours post-injection of LPS-GalN, and only LPS-GalN for 2 hours. Except for untreated control mice (Control), each mouse received 0.5 ml of LPS-GalN (consisting of 100 ng of LPS plus 8 mg of D-galactosamine i.p.). The depicted Whisker plots represent the median and values. In (A), the lung histopathological score (“lung injury score”) was graded according to a 5-point scale from 0 to 4 as follows: 0, l, 2, 3, and 4 represented no damage, mild damage, moderate damage, severe damage, and very severe damage, respectively. In (B), the liver histopathological score (“liver injury score”) was graded according to a 5-point scale from 0 to 4 as follows: 0, l, 2, 3, and 4 represented no damage, mild damage, moderate damage, severe damage, and very severe damage, respectively. Kruskal-Wallis test and Mann Whitney U test were used for comparing the results among different treatment groups. Statistical significance between groups is shown by # p<0.05; as compared to LPS/GaIN (2h kill) group, and $ p<0.05; $$ p<0.01; as compared to LPS/GaIN+NS group.

**Figure S3.** The Effects of Rejuveinix (RJX) and the different doses of the Dexamethasone (DEX), treatments on Acute Lung Injury and Inflammation in a Mouse Model of Fatal Cytokine Storm, Sepsis, Systemic Inflammation, ARDS and Multiorgan Failure. Groups of 6 BALB/C mice were treated with i.p injections of RJX (6-fold diluted, 4.2 mL/kg, 0.5 ml/mouse), DEX (0.1 mg/kg, 0.6 mg/kg, and 6.0 mg/kg, 0.5 mL/mouse), or vehicle (NS, 0.5 mL/mouse) two hours post-injection of LPS-GalN. Except for untreated control mice (Control, Panel A), each mouse received 0.5 ml of LPS-GalN (consisting of 100 ng of LPS plus 8 mg of D-galactosamine i.p.). The lung histopathological ALI scores were 0 for each of the control mice (Panel A), ranged from 3 to 4 (Median: 4) for the LPS-GalN+NS group (Panel B), from 2-3 (Median: 3) for LPS-GalN+RJX (0.7 ml/kg) group (Panel C), from 3-4 (Median: 3.5) for LPS-GalN+DEX (0.1 mg/kg) (Panel D), from 2-4 (Median: 3) for LPS-GalN+DEX (0.6 mg/kg) group (Panel E) and from 1-3 (Median: 2) for LPS-GalN+DEX (6.0 mg/kg) group (Panel F). Depicted are microscopic images of lung tissues of representative mice from the untreated control group and various treatment groups. White arrow: inflammatory cell infiltration; Black arrow (short): Exudate, edema; Black arrow (Long): hemorrhage; Black double-headed arrow: the thickness of the alveolar wall. H&E X400.

**Figure S4.** The Effects of Rejuveinix (RJX) and the different doses of the Dexamethasone (DEX), treatments on Liver Injury and Inflammation in a Mouse Model of Fatal Cytokine Storm, Sepsis, Systemic Inflammation, ARDS and Multiorgan Failure. Groups of 6 BALB/C mice were treated with i.p injections of RJX (6-fold diluted, 4.2 mL/kg, 0.5 ml/mouse), DEX (0.1 mg/kg, 0.6 mg/kg, and 6.0 mg/kg, 0.5 mL/mouse), or vehicle (NS, 0.5 mL/mouse) two hours post-injection of LPS-GalN. Except for untreated control mice (Control Panel A), each mouse received 0.5 ml of LPS-GalN (consisting of 100 ng of LPS plus 8 mg of D-galactosamine i.p.). The liver histopathological scores ranged from 3 to 4 for the LPS-GalN+NS group (Panel B), from 2-3 for LPS-GalN+RJX group (Panel C), from 3-4 for LPS-GalN+DEX (0.1) (Panel D), from 2-3 for LPS-GalN+DEX (0.6) group (Panel E) and from 2-3 for LPS-GalN+DEX (6.0) group (Panel F). Depicted are microscopic images of the liver tissues of representative mice from the untreated control group and various treatment groups. While the liver histopathological score median was 0 for untreated control mouse, the liver score median for the depicted mice treated with 0.6 mg/kg or 6.0 mg/kg DEX were 3 for each, and the liver score median for the LPS-GaIN+NS treated control mice was 3.5. Black arrow (short): Inflammatory cell infiltration; Black arrow (long): Congestion; White arrow (long): Necrosis; White arrow (short): Hydropic degeneration. H&E 200x

**Figure S5.** *In Vivo* Treatment Activity of Rejuveinix (RJX) and the different doses of the Dexamethasone (DEX) in the LPS-GalN Mouse Model of Fatal Cytokine Storm, Sepsis, Systemic Inflammation, ARDS and Multiorgan Failure. BALB/C mice were treated with i.p injections of RJX (4.2 mL/kg or 8.4 mL/kg of 1:6-diluted RX = 0.7 mL/kg or 1.4 mL/kg RJX; 0.5 ml/mouse), DEX (0.1 mg/kg, 0.6 mg/kg and 6.0 mg/kg 0.5 mL/mouse), or vehicle (NS, 0.5 mL/mouse) two hours post-injection of LPS-GalN. Except for untreated control mice (Control), each mouse received 0.5 ml of LPS-GalN (consisting of 100 ng of LPS plus 8 mg of D-galactosamine i.p.). The cumulative proportion of mice remaining alive (Survival, %) is shown as a function of time after the LPS-GalN challenge. Depicted are the Kaplan Meier survival curves (Panel A) and survival data with statistical analysis (Panels B and C) of the different treatment groups.

**Figure S6.** RJX (0.7 mL/kg) plus DEX (6.0 mg/kg) Combination Mitigates Acute Lung Injury and Inflammation in a Mouse Model of Fatal Cytokine Storm and Sepsis. [A]: Lung tissue of a representative mouse injected with LPS-GalN without any pre- or post-LPS-GalN treatments and electively sacrificed at 2 hours to confirm the rapid onset of lung damage. The histopathological ALI score was 3 consistent with severe lung damage. Yellow arrow: inflammatory cell infiltration; blue arrow: exudate; orange arrow: hemorrhage; green block: thickness of alveolar wall. [B] Lung tissue of a LPS-GalN injected representative mouse treated with a single dose of RJX at 2 hours post-LPS-GalN. The mouse was electively sacrificed at 24 hours post LPS-GalN. ALI score = 2 (moderate lung damage). [C] Lung tissue of a LPS-GalN injected representative mouse treated with a single dose of DEX at 2 hours post-LPS-GalN. The mouse was electively sacrificed at 24 hours post LPS-GalN. ALI score = 2 (moderate lung damage). [D] ] Lung tissue of a LPS-GalN injected representative mouse treated with a single dose of NS at 2 hours post-LPS-GalN. The mouse died of sepsis at 4.2 hours post LPS-GalN. ALI score = 4 (very severe lung damage). Yellow arrow: inflammatory cell infiltration; blue arrow: exudate; orange arrow: hemorrhage; green block: thickness of alveolar wall. [E] Lung tissue of a healthy control mouse not injected with LPS-GalN and electively sacrificed at 24 hours. ALI score = 0 (no lung damage). [F] & [G]. Lung tissues from two representative LPS-GalN injected control mice treated RJX + DEX 2 hours before at 2 hours post-LPS-GalN. These mice survived the LPS-GalN challenge and were electively sacrificed at 24 hours. No lung damage was detected (Histopathological lung score/ALI score = 0). [H] Lung tissue of a LPS-GalN injected representative mouse treated with RJX + DEX at 2 hours post-LPS-GalN. The mouse was electively sacrificed at 24 hours post LPS-GalN. ALI score = 1 (mild lung damage). H&E X400

**Figure S7.** Therapeutic Use of Low Dose RJX + Supratherapeutic High Dose DEX Combination After Onset of Systemic Inflammation and Lung Injury Improves the Survival Outcome in the LPS-GalN Mouse Model of Fatal Cytokine Storm and Sepsis. Groups of 6 BALB/C mice were treated with i.p injections of RJX (6-fold diluted, 4.2 mL/kg, 0.5 ml/mouse), DEX (6 mg/kg, 0.5 mL/mouse), RJX + DEX (0.5 mL/mouse), or vehicle (NS, 0.5 mL/mouse) two hours post-injection of LPS-GalN. Except for untreated control mice (Control), each mouse received 0.5 ml of LPS-GalN (consisting of 100 ng of LPS plus 8 mg of D-galactosamine) i.p. The depicted Whisker plots represent the median and values for time to death from 6 mice in each group. Kruskall Wallis and Mann Whitney U (pairwise comparisons test) test were used for comparing the results among different treatment groups. Statistical significance between groups is shown by: ** p<0.01 compared as control group and, # p<0.05; ## p<0.01 compared as LPS/GaIN+NS group.

**Figure S8. Time-dependent decrease of CRP Values in RJX-Treated Severe COVID-19 Patients.** Decay of Serum CRP concentration is depicted over 46 days for 9 evaluable responders (**A**). The first order kinetics of the reductions in serum Serum CRP values was investigated by fitting a straight line to a semi-log plot of the portion of the Serum CRP concentration-time curve that displayed maximum reduction in Serum CRP values over the course of RJX treatment (36 independent data points across 7 days) (**B**). The slope of the line represents the rate constant for Serum CRP reduction in log10 scale (-0.2231), and times to 50% reduction (T50) (1.53 days) of Serum CRP values were calculated using the rate constant and intercept value of 2.04.

**Figure S9. Swimmer Plot of CRP Normalization Kinetics in Severe COVID-19 Patients Treated with RJX Plus SOC.** Among the 9 patients who recovered after protocol therapy, the estimated time to reduction of baseline CRP values by 50% ranged from 0.9 days to 2.5 days (Median: 1.7 days; Mean±SE = 1.6 ± 0.2 days).

**Figure S10. Time-dependent Reduction of Serum IL-6 Concentration in RJX-Treated Severe COVID-19 Patients.** Decay of IL-6 concentration is depicted over 46 days for 5 evaluable responders (**A**). The first order kinetics of the reductions in serum IL-6 values was investigated by fitting a straight line to a semi-log plot of the portion of the IL-6 concentration-time curve that displayed maximum reduction in IL-6 values over the course of RJX treatment (18 independent data points across 7 days) (**B**). The slope of the line represents the rate constant for IL-6 reduction in log10 scale (-0.1698), and times to 50% reduction (T50) (2.51 days) of IL-6 values were calculated using the rate constant and intercept value of 2.13. Decay curves were also fitted to each of the 5 evaluable individual responders to calculate mean and standard error of the T50 parameter (Mean: 13.08 ± 6.13 days, Median: 6.3 days, Range: 1.62 - 29.31 days; For 2 patients T50>25 days, for 3 patients T50 < 7days).

**Figure S11. Time-dependent Reduction of Serum Ferritin Concentration in RJX-Treated Severe COVID-19 Patients.** Decay of Ferritin concentration is depicted over 46 days for 8 evaluable responders (**A**). The first order kinetics of the reductions in serum Ferritin values was investigated by fitting a straight line to a semi-log plot of the portion of the Ferritin concentration-time curve that displayed maximum reduction in Ferritin values over the course of RJX treatment (32 independent data points across 7 days) (**B**). The slope of the line represents the rate constant for Ferritin reduction in log10 scale (-0.0511), and times to 50% reduction (T50) (5.94 days) of Ferritin values were calculated using the rate constant and intercept value of 2. Decay curves were also fitted to each of the 8 evaluable individual responders to calculate mean and standard error of the T50 parameter (Mean: 8.83 ± 3.43 days, Median: 5.24 days, Range: 2.9 - 32.1 days).

**Figure S12. Time-dependent decrease of Serum LDH Values (in U/L) in RJX-Treated Severe COVID-19 Patients.** Decay of LDH concentration is depicted over 45 days for 5 evaluable responders (**A**). The first order kinetics of the reductions in serum LDH values was investigated by fitting a straight line to a semi-log plot of the portion of the LDH concentration-time curve that displayed maximum reduction in LDH values over the course of RJX treatment (20 independent data points across 7 days) (**B**). The slope of the line represents the rate constant for LDH reduction in log10 scale (-0.0511), and times to 50% reduction (T50) (6.86 days) of LDH values were calculated using the rate constant and intercept value of 2.05. Decay curves were also fitted to each of the 5 evaluable individual responders to calculate mean and standard error of the T50 parameter (Mean: 8.63 ± 1.88 days, Median: 7.22 days, Range: 4.87 - 14.55 days)

**Figure S13. Time-dependent decrease of Serum TGF-β Values in RJX-Treated Severe COVID-19 Patients.** Decay of TGF**-**β concentration is depicted over 45 days for 5 evaluable responders (**A**). The first order kinetics of the reductions in serum TGF**-**β values was investigated by fitting a straight line to a semi-log plot of the portion of the TGF**-**β concentration-time curve that displayed reduction of TGF**-**β values over the course of RJX treatment (33 independent data points across 45 days) (**B**). The slope of the line represents the rate constant TGF**-**β reduction in log10 scale (-0.0055), and times to 50% reduction (T50) (55.48 days) of TGF**-**β values were calculated using the rate constant and intercept value of 2.01. Decay curves were also fitted to each of the 4 evaluable individual responders to calculate mean and standard error of the T50 parameter (Mean: 26.56 ± 17.32 days, Median: 13.16 days, Range: 2.33 - 77.57 days)

**Figure S14.** **Survival Outcome of Severe COVID-19 Patients Treated with RJX Plus SOC.** Depicted is the overall survival curve of the 12 hospitalized COVID-19 patients along with the corresponding censor plot. All patients were treated with RJX + institutional SOC. Nine patients recovered and remained alive for ≥60 days.

**Figure S15.** Swimmer plot of clinical health score changes in RJX treated COVID-19 patients. Scores according to the 8-point ordinal scale are indicated with different colors. Arrow: Alive.

**Figure S16. A Waterfall plot of Maximum Clinical Score Change on 8-Point Ordinal Scale**
