# Supplementary material for: Evaluation of the potential of Rejuveinix plus dexamethasone against sepsis
Source: Future Microbiol. 2022 Sep 2:10.2217/fmb-2022-0044. doi: 10.2217/fmb-2022-0044 (PMC9443789; doi:10.2217/fmb-2022-0044)
Supplement: Supplementary file 1 [file supplementary_material.zip › Table_S1.docx]

**Table S1. Patient Characteristics and Demographic Features for Part 1 of RPI015 Study**

| **UPN** | **CH#** | **Age/Sex/Race** | **Co-morbidities** | **Ethnicity** | **Height (in cm)/Weight (in kg)/**  **BMI (in kg/m^2^)** | **Hx of Vac./**  **Covid-19 Dx.** | **8-point**  **Ordinal Scale^c^** | **Pneumonia Findings**  **CxR/CT** | **CRP**  **mg/L** | **ALC**  **(10^9^/L)** | **Fever/Cough/**  **SOB/Hypoxia**** | **Dx-ICF** |
| --- | --- | --- | --- | --- | --- | --- | --- | --- | --- | --- | --- | --- |
| 002-1102 | 1 | 35/Male/White | Obesity | H/L | 167.6/102.8/36.6 | -/+^a^ | 4 | + | 64.3 | 0.9 | +/+/+/+ | 8 |
| 002-1203 | 2 | 45/Male/White | Obesity | H/L | 170.2/93.0/32.1 | -/+^a^ | 3 | + | 152 | 0.5 | +/+/+/+ | 1 |
| 002-1104 | 1 | 67/Male/White | Obesity, HTN,DM,HC | H/L | 172.7/104.8/35.1 | +/+^a^ | 4 | + | 55.3 | 1.4 | -/+/+/+ | 2 |
| 008-1201 | 2 | 60/Male/Asian | - | Not H/L | 167.6/66.7/23.8 | -/+^b^ | 3 | + | 80.3 | 0.4 | +/+/+/+ | 11 |
| 008-1103 | 1 | 54/Female/White | Obesity, HTN,Asthma | H/L | 157.5/90.7/36.6 | -/+^a^ | 4 | + | 94.6 | 0.5 | +/+/+/+ | 10 |
| *008-1204*  *(withdrew consent on D4)* | 2 | 46/Male/White | Obesity | H/L | 167.7/104.0/37.0 | -/+^a^ | 3 | + | 297.6 | 0.5 | +/+/+/+ | 6 |
| 008-1105 | 1 | 48/Male/White | Obesity, HTN, DM | H/L | 177.8/156.0/49.4 | -/+^a^ | 4 | + | 106.5 | 0.3 | +/+/+/+ | 7 |
| 008-1208 | 2 | 43/Male/White | Obesity, CHF, COPD | H/L | 162.6/89.4/33.8 | -/+^a^ | 3 | + | 42.4 | 1.2 | +/+/+/+ | 4 |
| 008-1210 | 2 | 41/Female/White | Obesity Asthma | H/L | 167.6/115.0/40.9 | +/+^a^ | 3 | + | 54.1 | 0.9 | +/+/+/+ | 6 |
| 007-1201 | 2 | 72/ Male/White | Overweight, HTN | H/L | 165.1/77.1/28.3 | -/+^a^ | 3 | + | 50.3 | 0.5 | -/+/+/+ | 1 |
| 007-1202 | 2 | 70/Female/White | Obesity, HTN,DM | H/L | 152.4/77.1/33.2 | -/+^a^ | 3 | + | 54.1 | 0.9 | -/-/+/+ | 0 |
| 008-1111 | 1 | 24/Female/Asian | Obesity | Not H/L | 160.0/118/46.1 | -/+^a^ | 4 | + | 53.4 | 0.9 | +/+/+/+ | 2 |
| 007-1103 | 1 | 45/Female/White | Overweight | H/L | 149.9/65.8/29.3 | -/+^a^ | 4 | + | 87.9 | 0.9 | -/+/+/+ | 1 |

All Cohort 2 patients had HRF requiring high-flow oxygen +/- non-invasive positive pressure ventilation (NIPPV). No patient on invasive mechanical ventilation (IMV) was eligible for this study. All Cohort 1 patients had hypoxia in room air requiring supplemental oxygen (not high-flow, NIPVV, or MV). Covid-19: Corona virus disease 2019; CT: Computed tomography; UPN: Unique patient number; CH: Cohort

^a^FDA-approved RT-PCR test ^b^ FDA-approved antigen test; ^c^8-point ordinal scale: 1. Death; 2. Hospitalized, on invasive mechanical ventilation or ECMO; 3. Hospitalized, on non-

invasive ventilation or high flow oxygen devices; 4. Hospitalized, requiring supplemental oxygen; 5. Hospitalized, not requiring supplemental oxygen - requiring ongoing medical care

(COVID-19 related or otherwise); 6. Hospitalized, not requiring; supplemental oxygen - no longer requires ongoing medical care; 7. Not hospitalized, limitation on activities and/or

requiring home oxygen; 8. Not hospitalized, no limitations on activities. HTN: hypertension; DM: diabetes mellitus; HC: hypercholesterolemia; H/L: Hispanic or Latino

**Patients 007-1201, 008-1102, 008-1103, 008-1210 also had diarrhea. Patient 008-1204 withdrew consent on day 4. Body Mass Index (BMI) was calculated using the formula: Weight(kg) / Height(m)^2^. In accordance with the federal guidelines on the identification, evaluation, and treatment of overweight and obesity in adults released by the National Heart, Lung, and Blood Institute (NHLBI), "overweight" was defined as a BMI value between 25 and 29.9; and "obesity" as a BMI value greater than or equal to 30.
