# Supplementary material for: Evaluation of the potential of Rejuveinix plus dexamethasone against sepsis
Source: Future Microbiol. 2022 Sep 2:10.2217/fmb-2022-0044. doi: 10.2217/fmb-2022-0044 (PMC9443789; doi:10.2217/fmb-2022-0044)
Supplement: Supplementary file 1 [file supplementary_material.zip › Table_S2.docx]

**Table S2. Concomitant Standard of Care Therapies**

| **Patient No.** | **Antiviral Therapy** | **Antibiotic Therapy** | **Anticoagulation** | **Non-steroidal Anti-Inflammatory Therapy** | **Corticosteroids** |
| --- | --- | --- | --- | --- | --- |
| 002-1102 | Remdesivir,  Convalescent plasma | Azithromycin, Ceftriaxone | Enoxaparin | - | Dexamethasone |
| 002-1203 | Remdesivir | Doxycycin, Ceftriaxone, Piperacillin-Tazobactam, | Enoxaparin | - | Dexamethasone, Solu-Medrol |
| 002-1104 | Remdesivir | Azithromycin, Ceftriaxone | Enoxaparin, Rivaroxaban | - | Dexamethasone |
| 008-1201 | Remdesivir | Azithromycin, Ceftriaxone | Enoxaparin | - | Dexamethasone, Budesonide |
| 008-1103 | - | Azithromycin, Ceftriaxone | Enoxaparin | - | Dexamethasone |
| 008-1204 | Remdesivir | Azithromycin, Ceftriaxone | Enoxaparin | - | Dexamethasone |
| 007-1103 | Acyclovir, Gancyclovir | Ertapenem | Enoxaparin | - | Solu-Medrol |
| 008-1105 | - | Piperacillin-Tazobactam,  Ceftriaxone | Enoxaparin | - | Dexamethasone |
| 008-1208 | Remdesivir | Azithromycin, Ceftriaxone | Enoxaparin | Ibuprofen | Dexamethasone |
| 008-1210 | Remdesivir | Azithromycin, Ceftriaxone, Linezolid, | Enoxaparin | - | Dexamethasone, Budesonide |
| 007-1201 | - | Ertapenem | Enoxaparin | - | Solu-medrol |
| 007-1202 | - | - | Enoxaparin | - | Solu-medrol, Prednisone |
| 008-1111 | - | Azithromycin, Ceftriaxone | Enoxaparin | - | Dexamethasone |
