# Supplementary material for: Evaluation of the potential of Rejuveinix plus dexamethasone against sepsis
Source: Future Microbiol. 2022 Sep 2:10.2217/fmb-2022-0044. doi: 10.2217/fmb-2022-0044 (PMC9443789; doi:10.2217/fmb-2022-0044)
Supplement: Supplementary file 1 [file supplementary_material.zip › Table_S3.docx]

| **Table S3: Listing of all Grade 3-5 AEs by MedDRA PT: All enrolled patients in Part 1 of the RPI015 study** | | | | | | | | | | | | | | |
| --- | --- | --- | --- | --- | --- | --- | --- | --- | --- | --- | --- | --- | --- | --- |
| **UPN** | **CH#** | **Preferred term** | **AE term (CTCAE Grade)** | **SAE** | **Date of onset (Number of days from ICF)** | **Date of resolution (Number of days from ICF)** | **Duration (days)** | **Relatedness to RJX** | **Relatedness to Covid-19** | **Action Taken with RJX** | **Outcome** | **DLT (Yes/No)** | **SUSAR (Yes/No)** | **PDC of RJX** |
| 002-1203 | Cohort 2 | Acute respiratory failure | Worsening acute hypoxemic respiratory failure (5) | Yes | Day 12 | NA | 15 (CODOD) | No | Yes | NA | NR, Fatal | No | No | No |
| 007-1201 | Cohort 2 | Sepsis | Sepsis (3) | No | Day 10 | NA | 14 (CODOD) | No | Yes | NA | NR | No | No | No |
|  |  | Fibrin D dimer increased | Elevated D-dimer (3) | No | Day 17 | NA | 7 (CODOD) | No | Yes | NA | NR | No | No | No |
|  |  | Pulmonary embolism | Acute pulmonary embolism right lower lobe (3) | No | Day 19 | NA | 5 (CODOD) | No | Yes | NA | NR | No | No | No |
|  |  | Cardiac arrest | Cardiac arrest (5) | Yes | Day 23 | NA | 1 (CODOD) | No | Yes | NA | NR, Fatal | No | No | No |
|  |  | Intestinal ischemia | Mesenteric ischemia (5) | Yes | Day 23 | NA | 1 (CODOD) | No | Yes | NA | NR, Fatal | No | No | No |
| 008-1210 | Cohort 2 | Acute respiratory failure | Worsening of acute hypoxic respiratory failure (5) | Yes | Day 8 | NA | 6  (CODOD) | No | Yes | NA | NR, Fatal | No | No | No |
|  |  | Multiple organ dysfunction syndrome | Multi organ failure (5) | Yes | Day 13 | NA | 1  (CODOD) | No | Yes | NA | NR, Fatal | No | No | No |
| AE: Adverse event; CH: Cohort; Covid-19: Corona virus disease 2019; CODOD: Censored on date of death; CTCAE: Common terminology criteria for adverse events; DLT: Dose limiting toxicity; ICF: Informed consent form; MedDRA: Medical dictionary for regulatory activities; NA: Not applicable; NR: Not recovered/not resolved; PD: Permanent discontinuation; RJX: Rejuveinix; SAE: Serious adverse event; SUSAR: Suspected unexpected serious adverse reaction; UPN: Unique patient number | | | | | | | | | | | | | | |
