# Supplementary material for: Evaluation of the potential of Rejuveinix plus dexamethasone against sepsis
Source: Future Microbiol. 2022 Sep 2:10.2217/fmb-2022-0044. doi: 10.2217/fmb-2022-0044 (PMC9443789; doi:10.2217/fmb-2022-0044)
Supplement: Supplementary file 1 [file supplementary_material.zip › Table_S5.docx]

| **Table S5: Incidence of Grade 3-5 AEs and SAEs by MedDRA PT and Listing of Deaths: All enrolled patients in Part 1 of the RPI015 study**   1. **Incidence of all Grade 3-5 AE** | | | |
| --- | --- | --- | --- |
| **MedDRA PT** | **Cohorts** | | **Total** |
|  | **Cohort 1 (N=6)** | **Cohort 2 (N=7)** | **N = 13 n (%)** |
| Acute respiratory failure | 0 | 2 (15.4%) | 2 (15.4%) |
| Cardiac arrest | 0 | 1 (7.7%) | 1 (7.7%) |
| Fibrin D dimer increased | 0 | 1 (7.7%) | 1 (7.7%) |
| Intestinal ischaemia | 0 | 1 (7.7%) | 1 (7.7%) |
| Multiple organ dysfunction syndrome | 0 | 1 (7.7%) | 1 (7.7%) |
| Pulmonary embolism | 0 | 1 (7.7%) | 1 (7.7%) |
| Sepsis | 0 | 1 (7.7%) | 1 (7.7%) |

1. **Incidence of all SAEs**

| **MedDRA PT** | **Cohorts** | | **Total** |
| --- | --- | --- | --- |
|  | **Cohort 1 (N=6)** | **Cohort 2 (N=7)** | **N = 13 n (%)** |
| Acute respiratory failure | 0 | 2 (15.4%) | 2 (15.4%) |
| Cardiac arrest | 0 | 1 (7.7%) | 1 (7.7%) |
| Intestinal ischaemia | 0 | 1 (7.7%) | 1 (7.7%) |
| Multiple organ dysfunction syndrome | 0 | 1 (7.7%) | 1 (7.7%) |

1. **Listing of Deaths**

| 1. Patient No. | Cohort# | Timing (Days) from First and Last RJX Infusion | Cause of death | Relatedness to RJX | Relatedness to COVID-19 |
| --- | --- | --- | --- | --- | --- |
| 002-1203 | Cohort 2 | 25 / 19 | SAE: Worsening Acute Hypoxemic Respiratory Failure | No | Yes |
| 007-1201 | Cohort 2 | 21 / 15 | SAE: Cardiac Arrest and Mesenteric Ischemia | No | Yes |
| 008-1210 | Cohort 2 | 14 / 8 | SAE: Worsening Acute Hypoxemic Respiratory Failure ; Multi-Organ Failure | No | Yes |
| RJX: Rejuveinix; SAE: Serious adverse event; MedDRA: Medical dictionary for regulatory activities; N: Total number of patients; n: Number of patients with event; PT: Preferred term | | | | | |
