# Supplementary material for: Evaluation of the potential of Rejuveinix plus dexamethasone against sepsis
Source: Future Microbiol. 2022 Sep 2:10.2217/fmb-2022-0044. doi: 10.2217/fmb-2022-0044 (PMC9443789; doi:10.2217/fmb-2022-0044)
Supplement: Supplementary file 1 [file supplementary_material.zip › Table_S6.docx]

| **Table S6: Treatment Outcome of Patients in Part 1 of RPI015 Study** | | | | | | | | | | | | |  |
| --- | --- | --- | --- | --- | --- | --- | --- | --- | --- | --- | --- | --- | --- |
| **Patient No.** | **Cohort#** | **Progression of COVID-19 and NIV Failure** | **Required IMV** | **Time to IMV (Time to death)** | **Clinical Improvement** | | **CXR/CT Improvement at Discharge from Hospital** | **Recovery on 8-point Ordinal Scale** |  | **Patient status at the last follow-up date** | | | |
|  |  |  |  |  | **Time from 1^st^ dose of RJX to Resolution of HRF**  **(Time to Discharge)**  **in Days** | **COVID-19 Symptoms Improved/**  **Resolved** |  |  |  | **Last follow-up date (Days from ICF)** | **Status (Alive/Dead)** | **ECOG Performance Status** | |
| 002-1102 | 1 | No | No | NA(NA) | NA (4) | +/+ | NA | +3 |  | 65 | Alive | 0 | |
| 002-1203 | 2 | Yes | Yes | 8 (26) | NA (NA) | -/- | NO | -2 (no recovery) |  | 26 | Dead | 5 | |
| 002-1104 | 1 | No | No | NA(NA) | NA (3) | +/+ | NA | +3 |  | 64 | Alive | 0 | |
| 008-1201 | 2 | No | No | NA(NA) | 5 (7) | +/+ | YES | +5 |  | 68 | Alive | 0 | |
| 008-1103 | 1 | No | No | NA(NA) | NA (5) | +/+ | YES | +3 |  | 65 | Alive | 0 | |
| 008-1105 | 1 | No | No | NA(NA) | NA (5) | +/+ | NO | +4 |  | 65 | Alive | 0 | |
| 008-1208 | 2 | No | No | NA (NA) | 3 (7) | +/+ | YES | +5 |  | 66 | Alive | 0 | |
| 008-1210 | 2 | Yes | Yes | 8 (14) | NA (NA) | -/- | NA | -2 (no recovery) |  | 14 | Dead | 5 | |
| 007-1201 | 2 | No* | No | 21 (21) | NA (NA) | -/- | NA | -2 (no recovery) |  | 21 | Dead | 5 | |
| 007-1202 | 2 | No | No | NA (NA) | 14 (14) | +/-^a^ | NA | +4 |  | 60 | Alive | 0 | |
| 008-1111 | 1 | No | No | NA (NA) | NA (3) | +/+ | NO | +3 |  | 65 | Alive | 0 | |
| 007-1103 | 1 | No | No | NA (NA) | NA (7) | +/+ | NA | +4 |  | 60 | Alive | 0 | |
| Covid-19: Corona virus disease 2019; NIV: Non-invasive ventilation; IMV: Invasive mechanical ventilation; ICF: Informed consent form; RJX: Rejuveinix; ^a^007-1202 reported mild residual  shortness of breath at last follow-up  8-point Ordinal Scale:  1. Death  2. Hospitalized, on invasive mechanical ventilation or ECMO  3. Hospitalized, on non-invasive ventilation or high flow oxygen devices  4. Hospitalized, requiring supplemental oxygen  5. Hospitalized, not requiring supplemental oxygen - requiring ongoing medical care (COVID-19 related or otherwise)  6. Hospitalized, not requiring supplemental oxygen - no longer requires ongoing medical care  7. Not hospitalized, limitation on activities and/or requiring home oxygen  8. Not hospitalized, no limitations on activities  ECOG Activity Level:  0 - Fully active, able to carry on all pre-disease performance without restriction; 1 - Restricted in physically strenuous activity but ambulatory and able to carry out work of a light or sedentary  nature, e.g., light house work, office work; 2 - Ambulatory and capable of all selfcare but unable to carry out any work activities. Up and about more than 50% of waking hours.  3 - Capable of only limited selfcare, confined to bed or chair more than 50% of waking hours; 4 - Completely disabled. Cannot carry on any selfcare. Totally confined to bed or chair.  5 - Dead. *007-1201: The HRF did not worsen but patient developed and died of mesenteric ischemia and cardiac arrest as Grade 5 SAEs.  Patient 008-1204 who withdrew his consent on day 4 is not shown in this table but included in safety analyses shown in Table S4 and Table S5. | | | | | | | | | | | | |  |
