# Supplementary figures and images for: Evaluation of the potential of Rejuveinix plus dexamethasone against sepsis
Source: Future Microbiol. 2022 Sep 2:10.2217/fmb-2022-0044. doi: 10.2217/fmb-2022-0044 (PMC9443789; doi:10.2217/fmb-2022-0044)

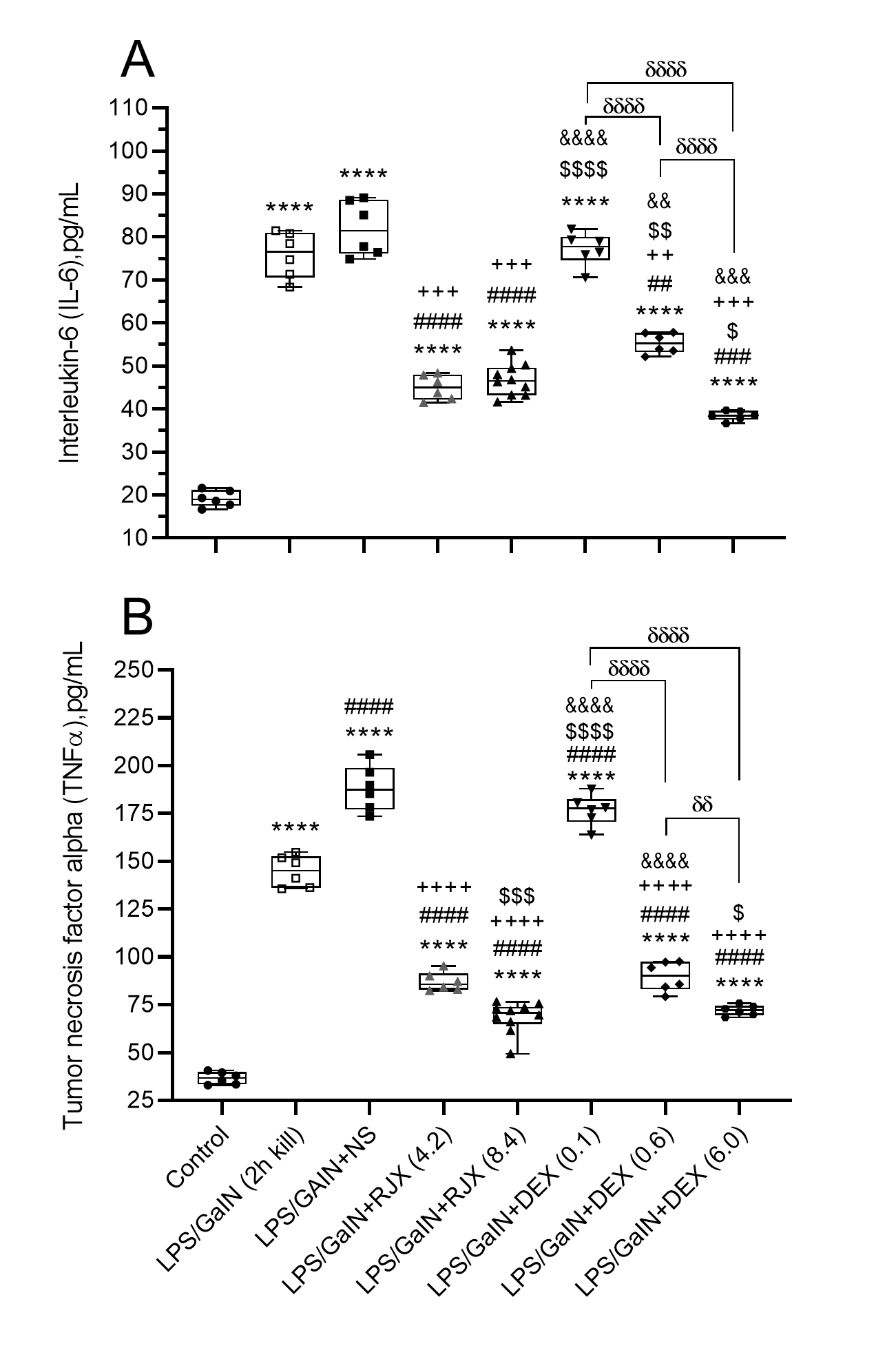

Supplement: Supplementary file 1 [file supplementary_material.zip › Fig_S1.tif]

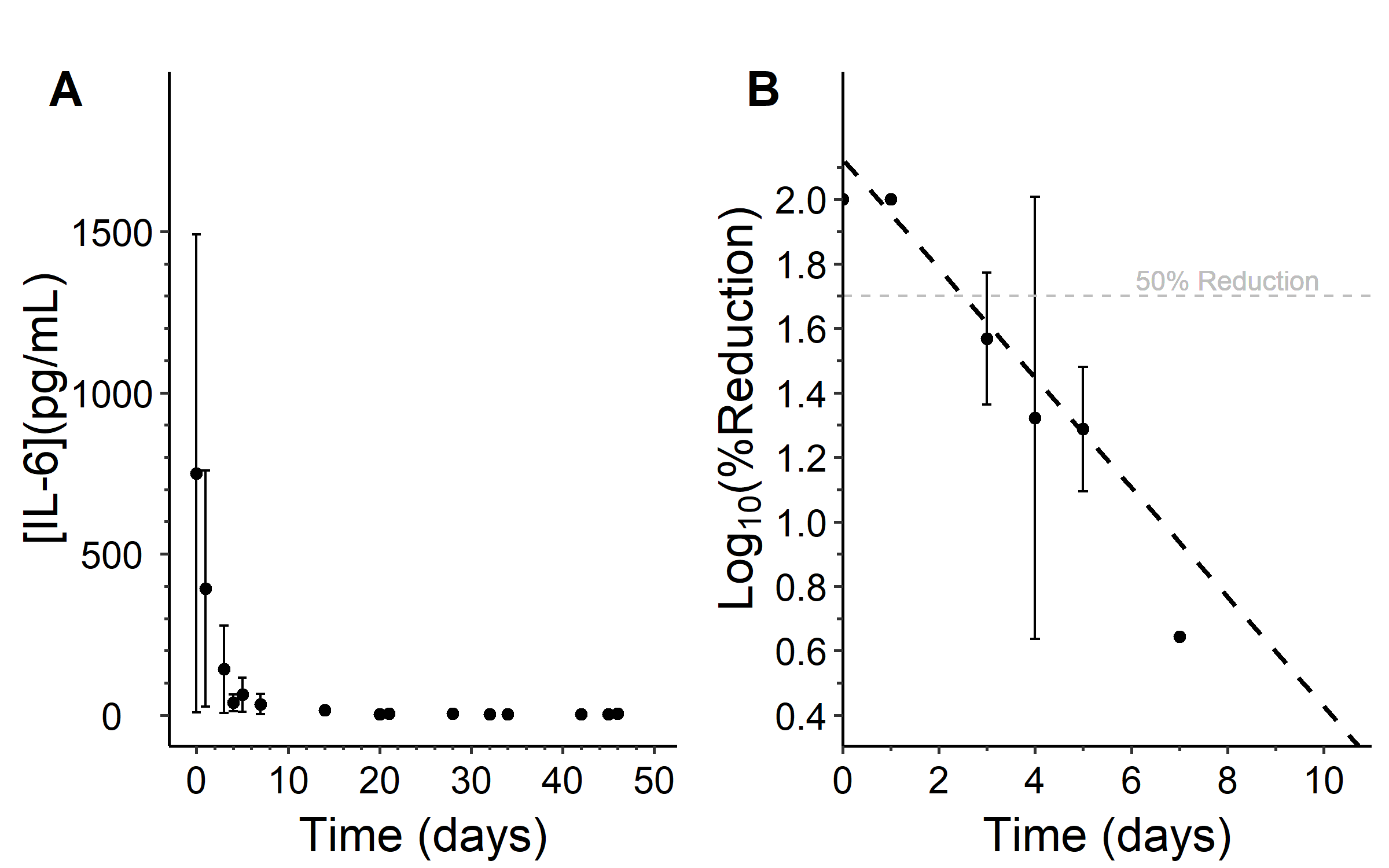

Supplement: Supplementary file 1 [file supplementary_material.zip › Fig_S10.tiff]

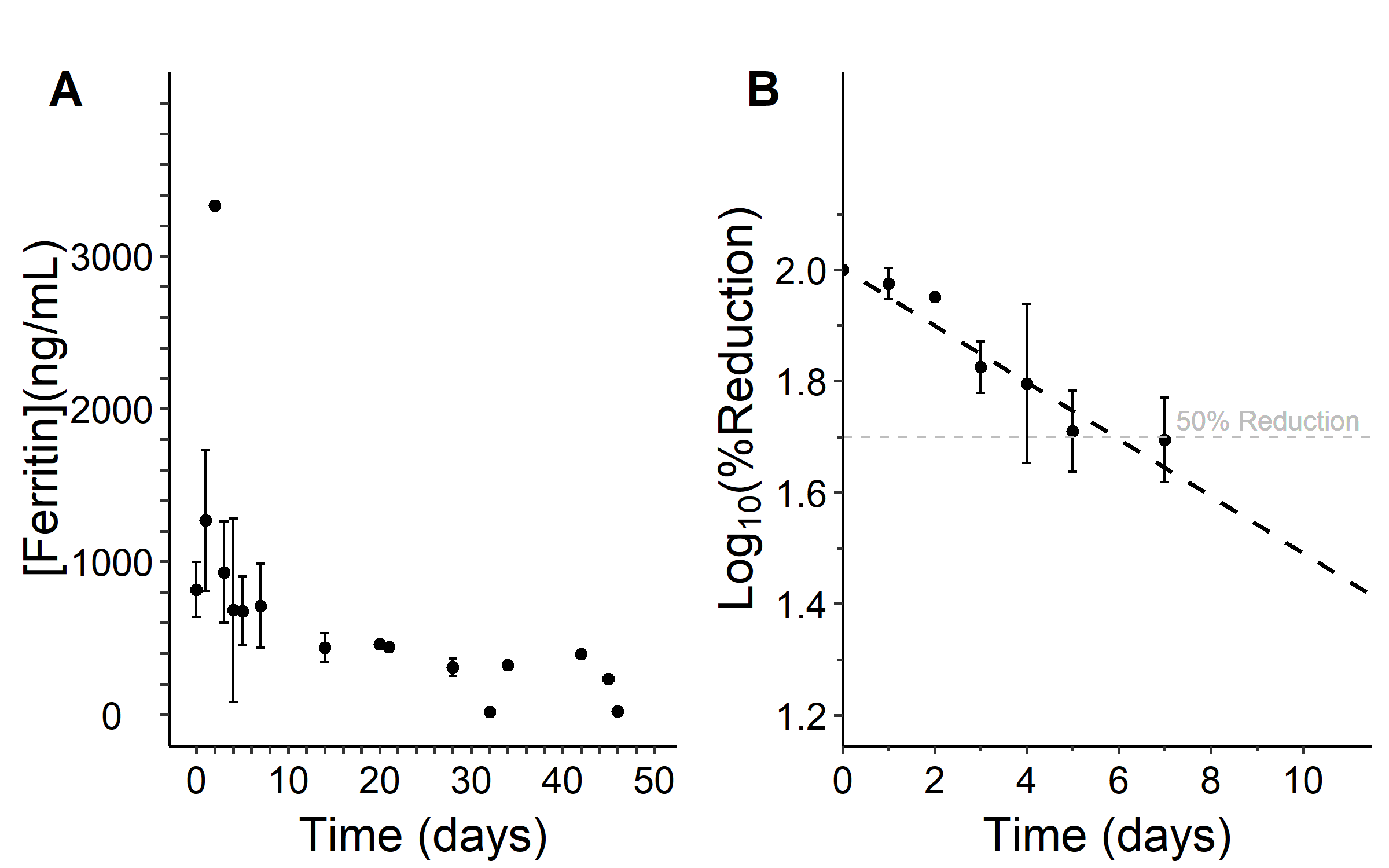

Supplement: Supplementary file 1 [file supplementary_material.zip › Fig_S11.tiff]

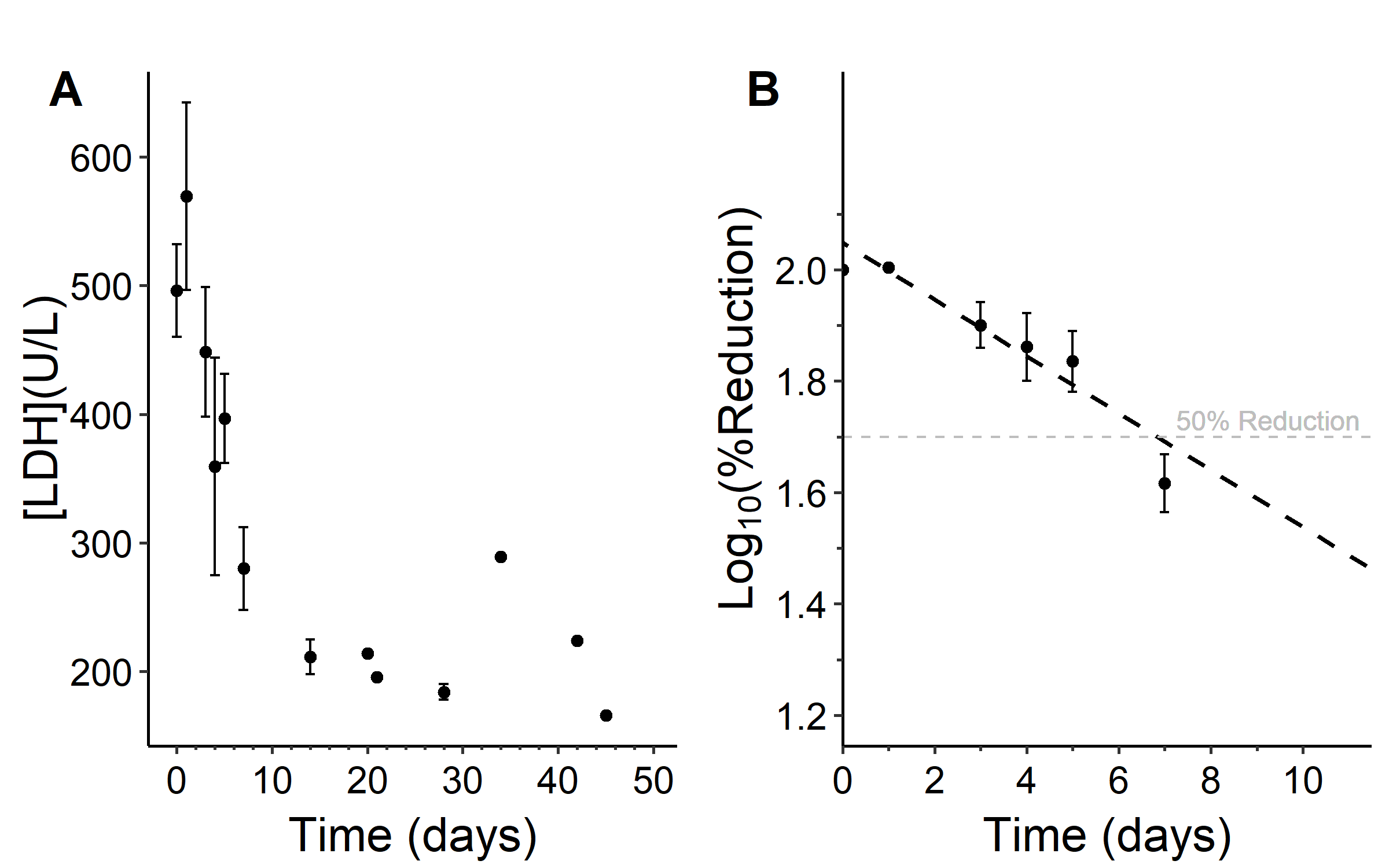

Supplement: Supplementary file 1 [file supplementary_material.zip › Fig_S12.tiff]

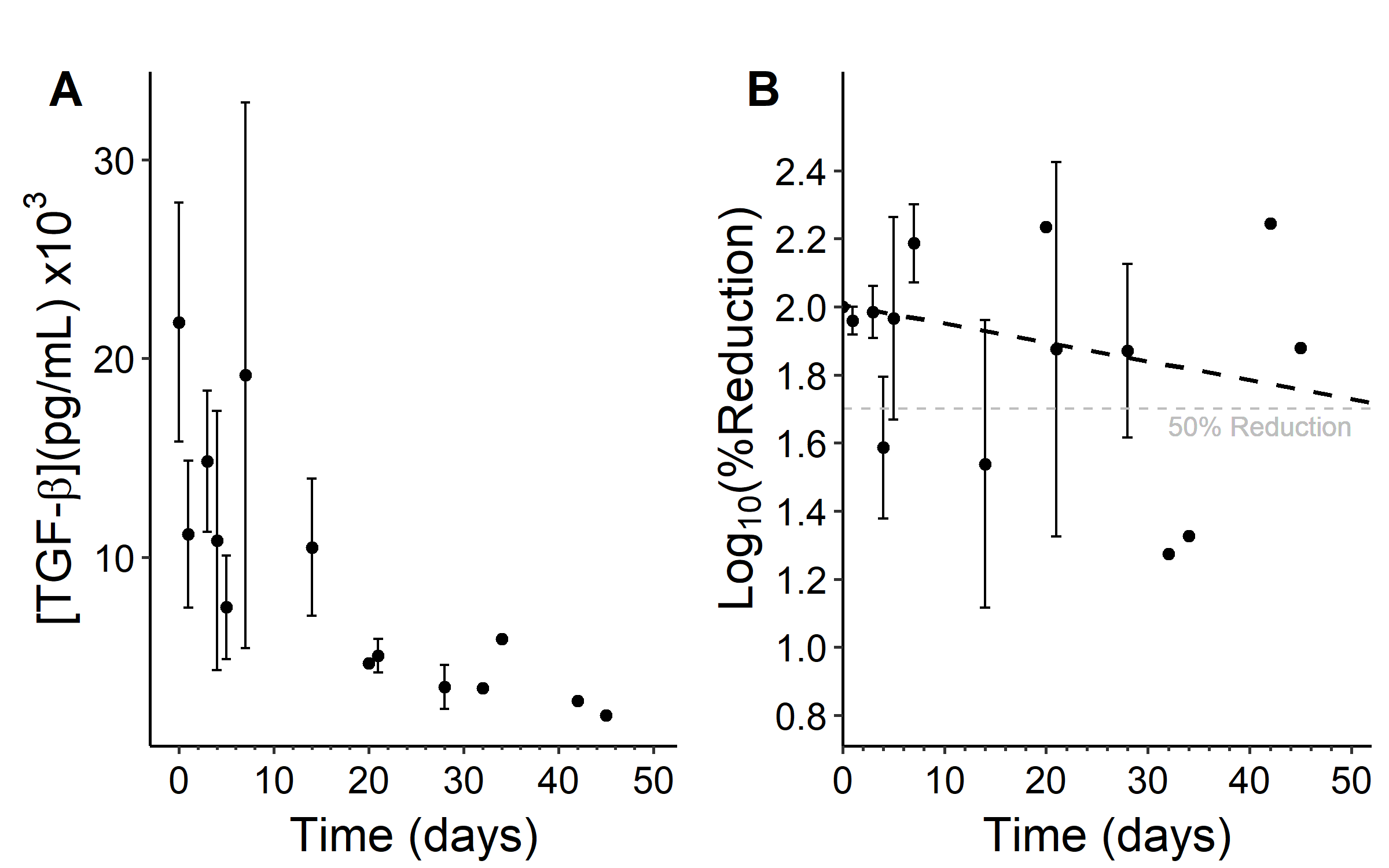

Supplement: Supplementary file 1 [file supplementary_material.zip › Fig_S13_TGFB_kinetic_rev.tiff]

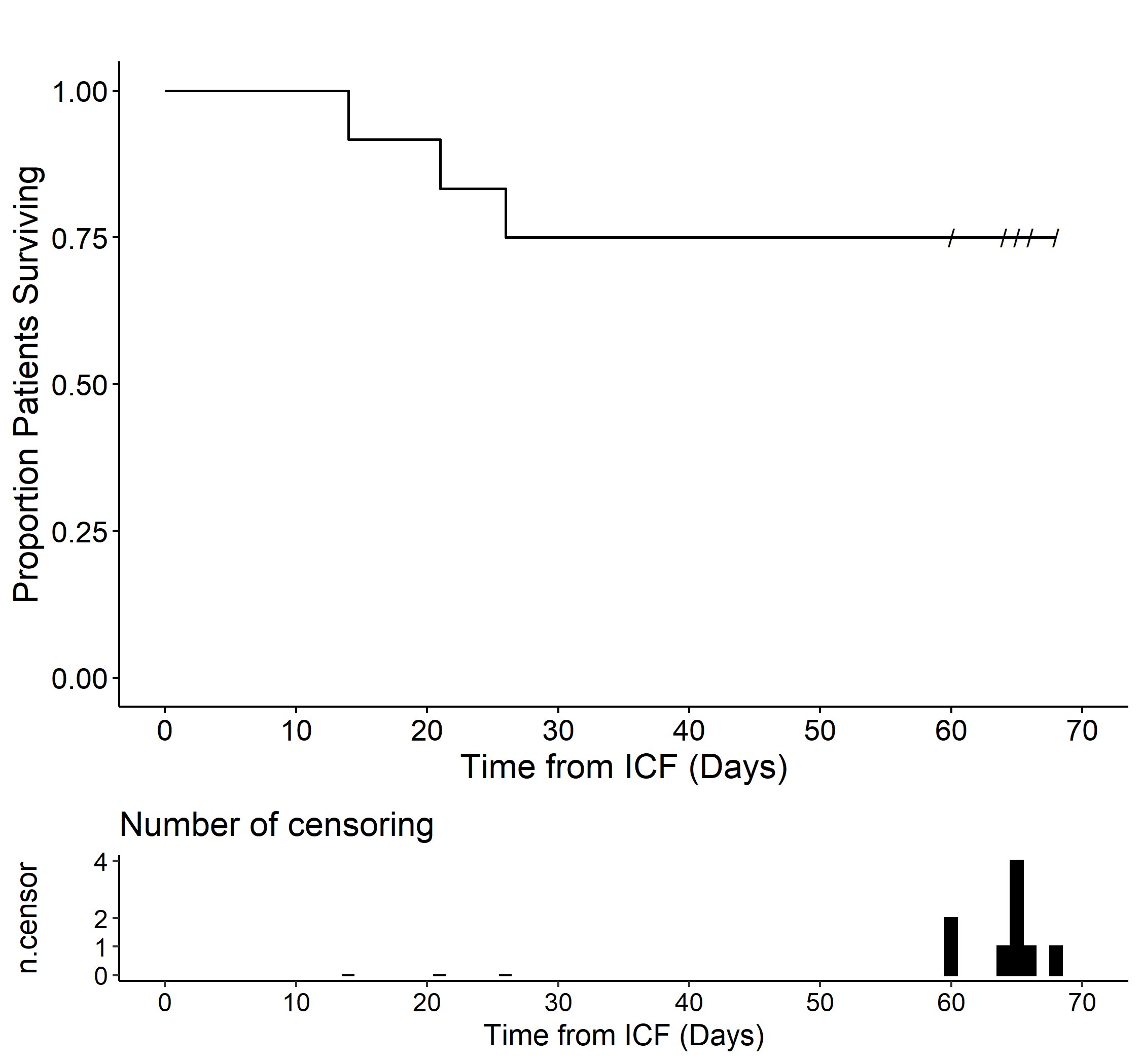

Supplement: Supplementary file 1 [file supplementary_material.zip › Fig_S14_OS.tiff]

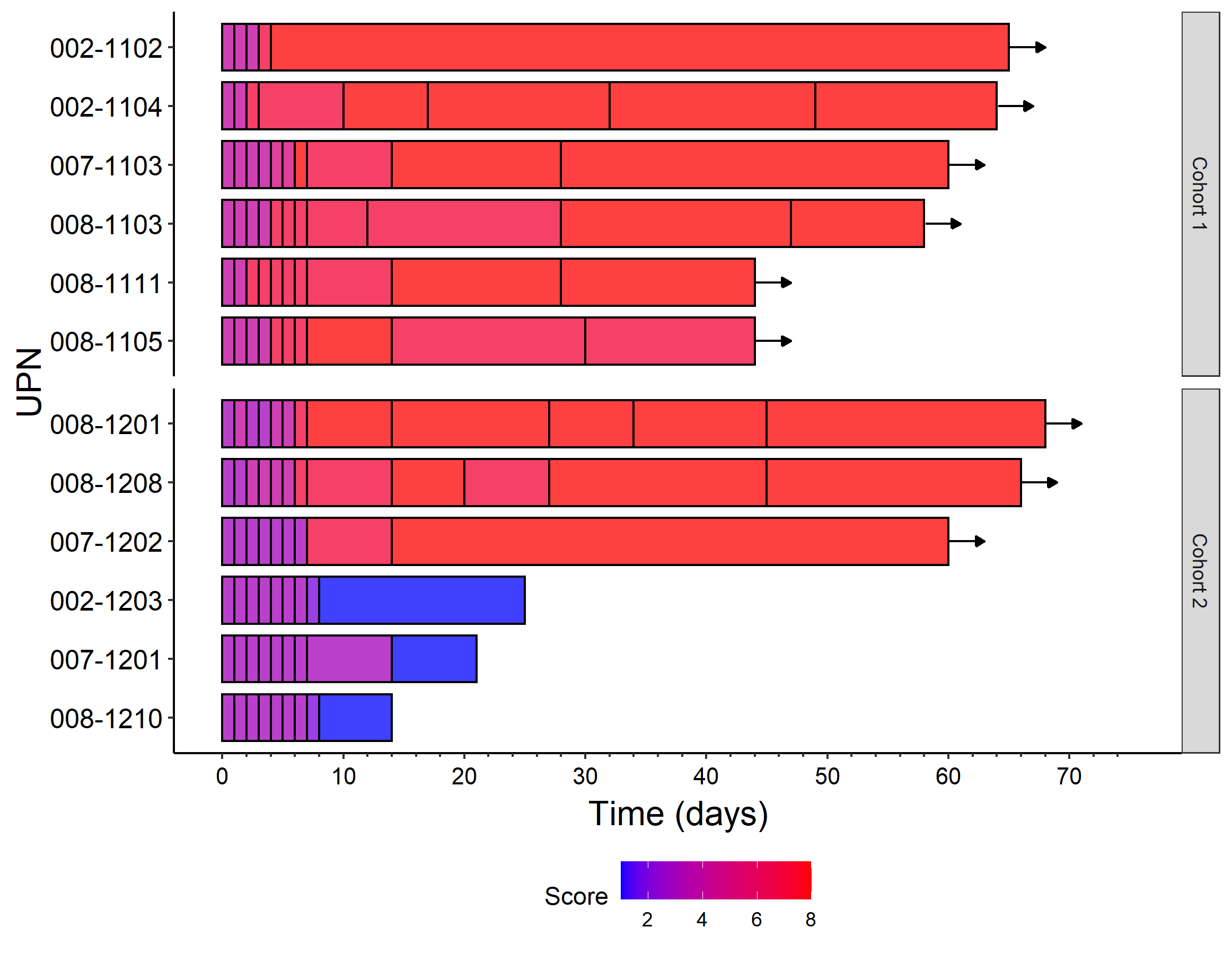

Supplement: Supplementary file 1 [file supplementary_material.zip › Fig_S15_swimmer_arrows.tiff]

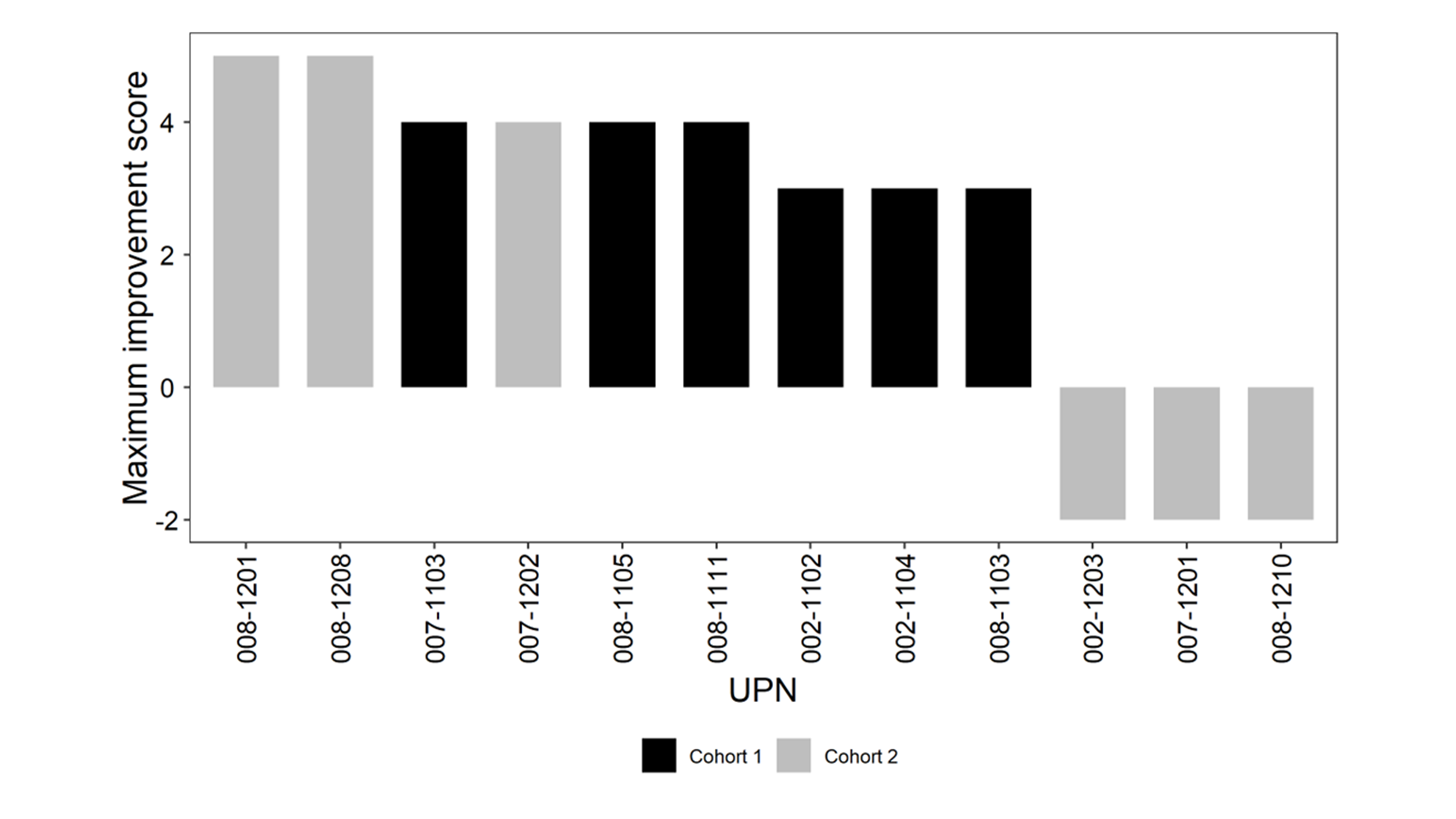

Supplement: Supplementary file 1 [file supplementary_material.zip › Fig_S16_Max_Improve.tif]

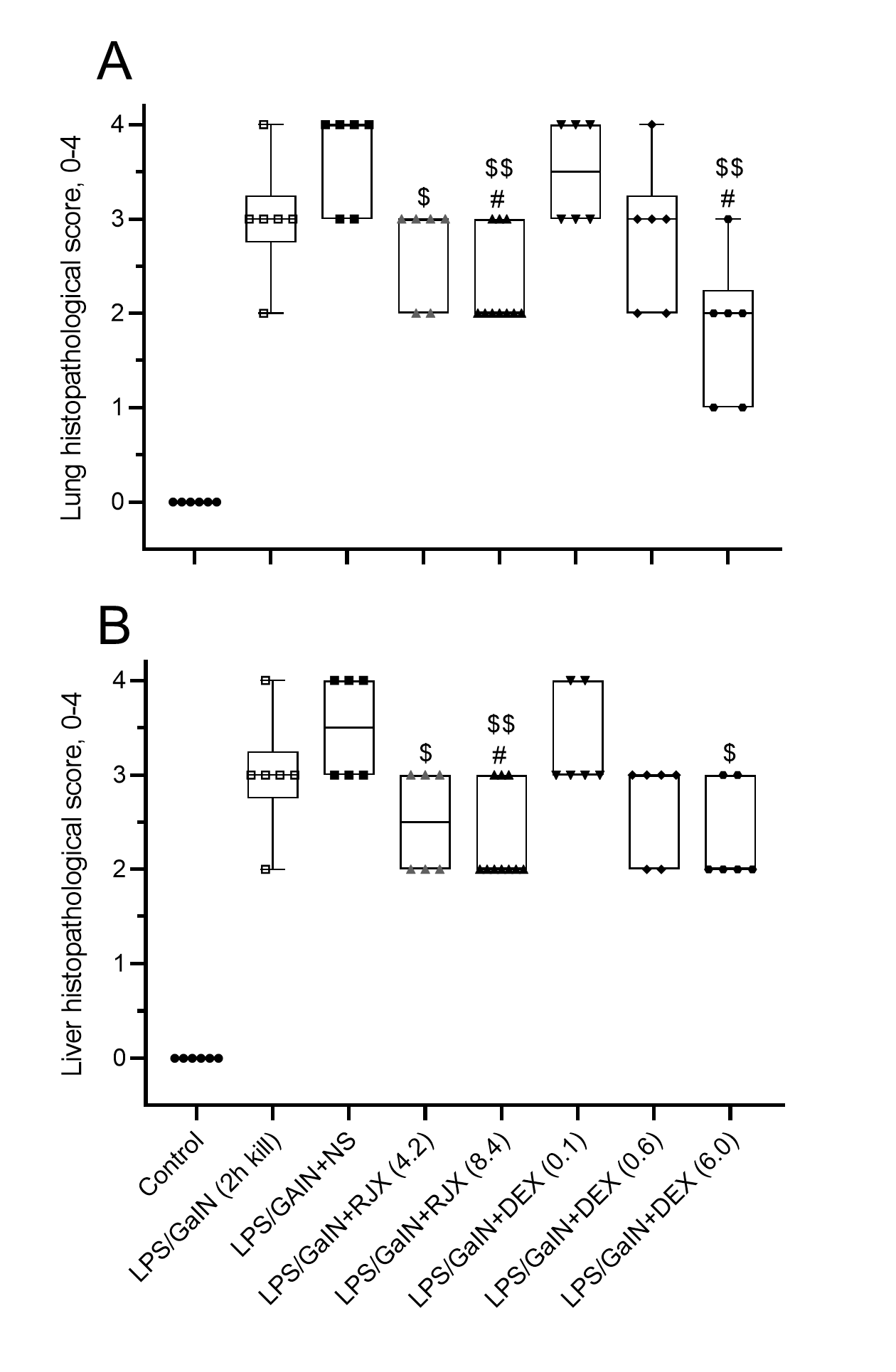

Supplement: Supplementary file 1 [file supplementary_material.zip › Fig_S2.tif]

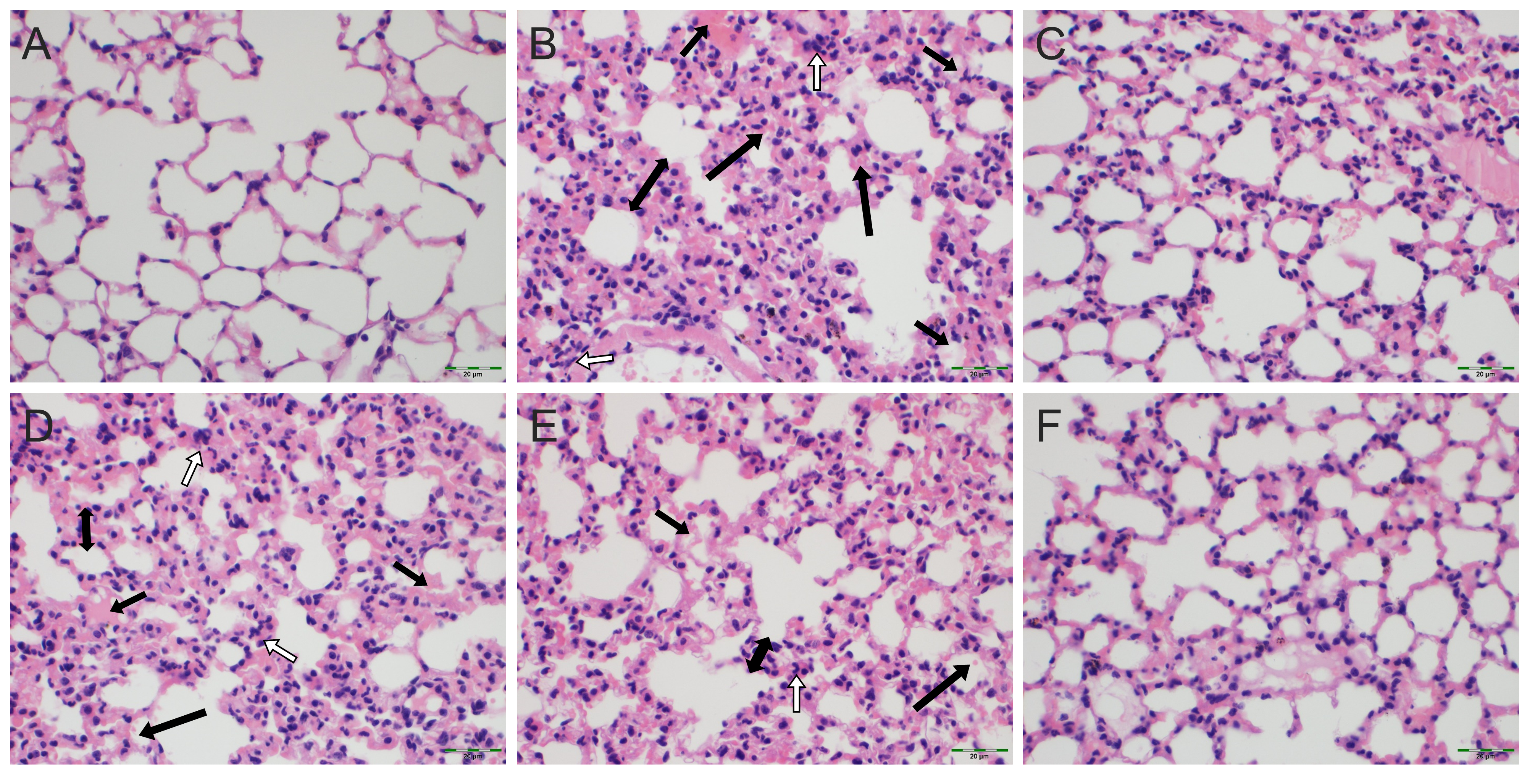

Supplement: Supplementary file 1 [file supplementary_material.zip › Fig_S3.tif]

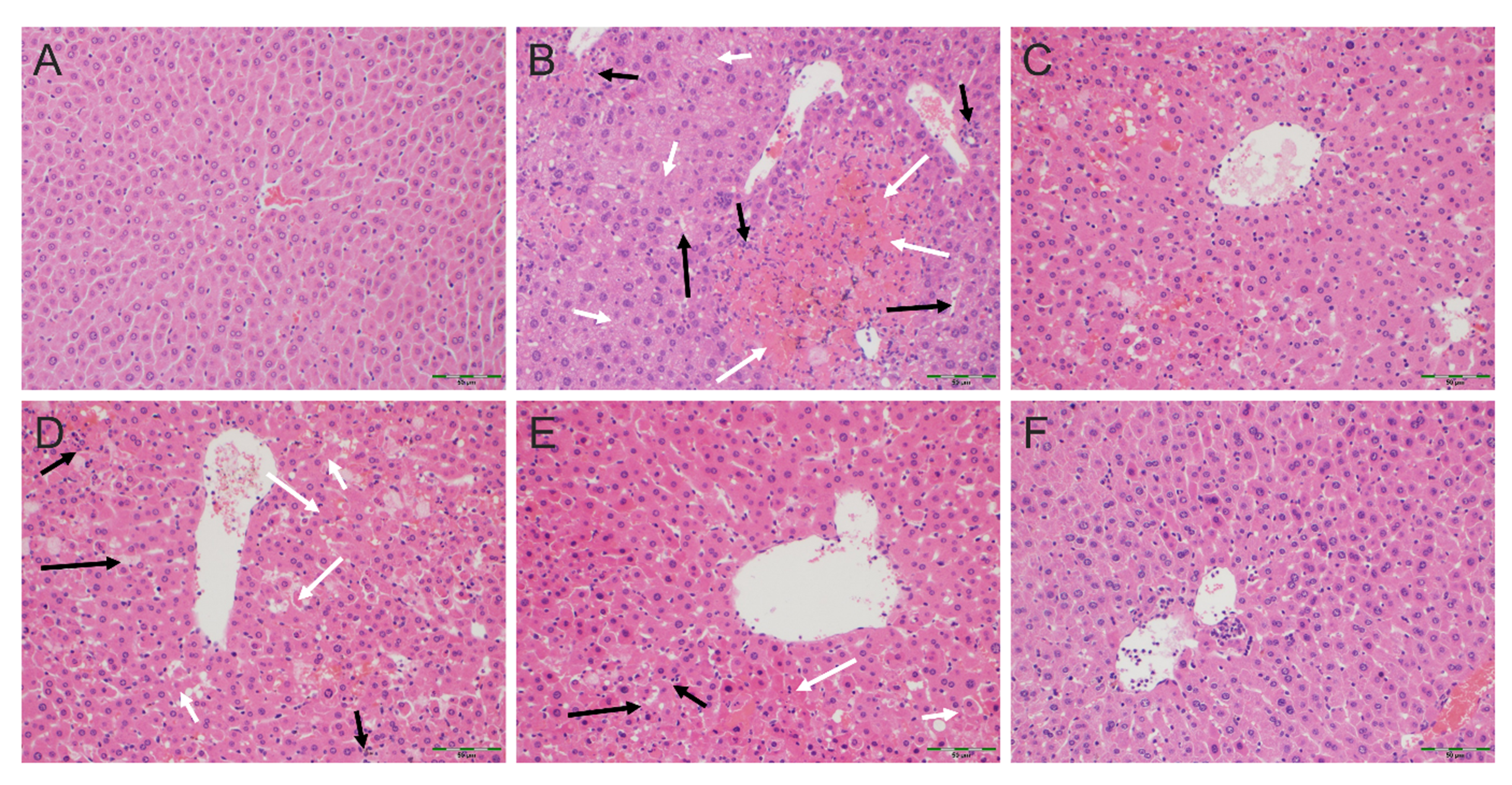

Supplement: Supplementary file 1 [file supplementary_material.zip › Fig_S4.tif]

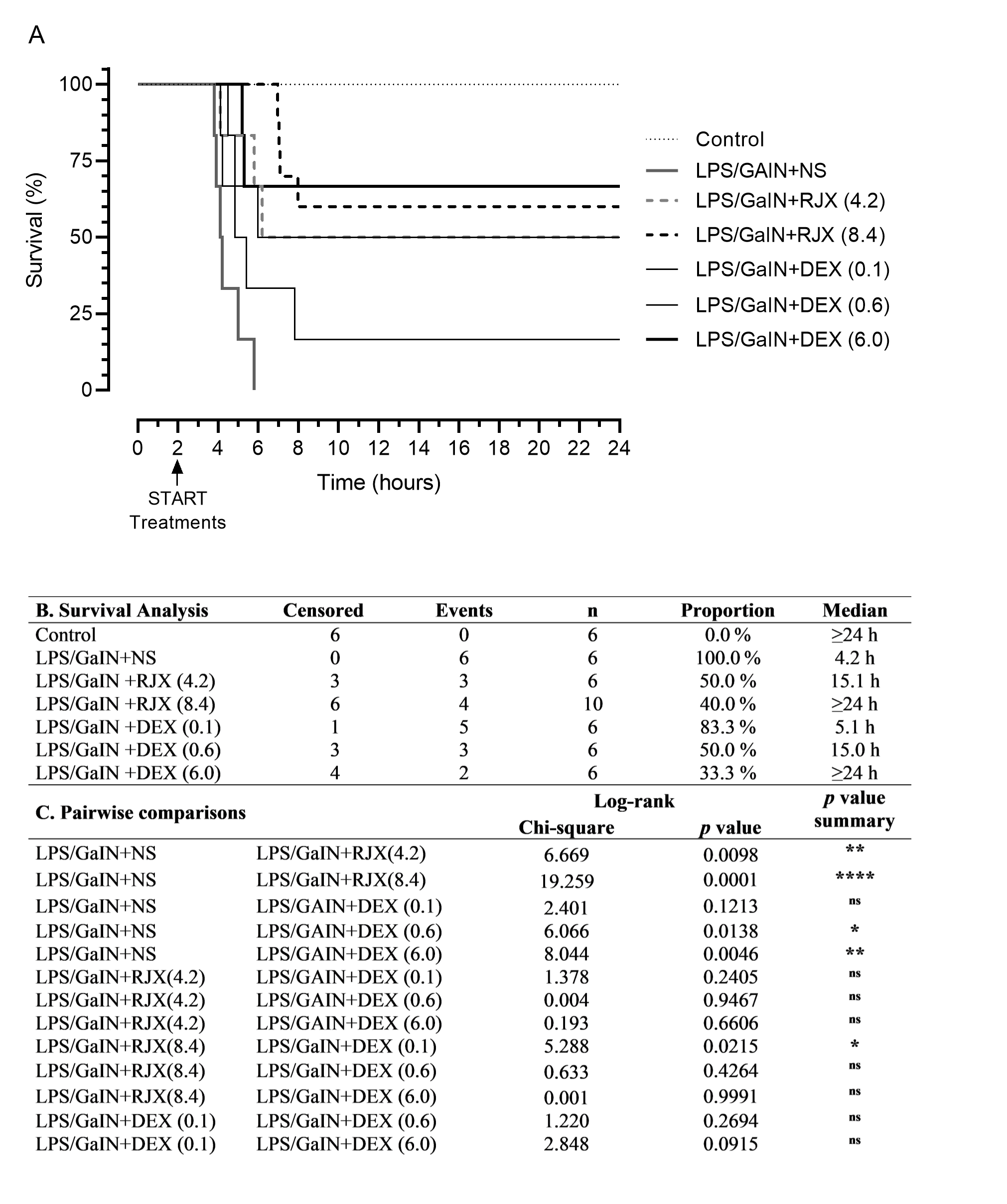

Supplement: Supplementary file 1 [file supplementary_material.zip › Fig_S5.tif]

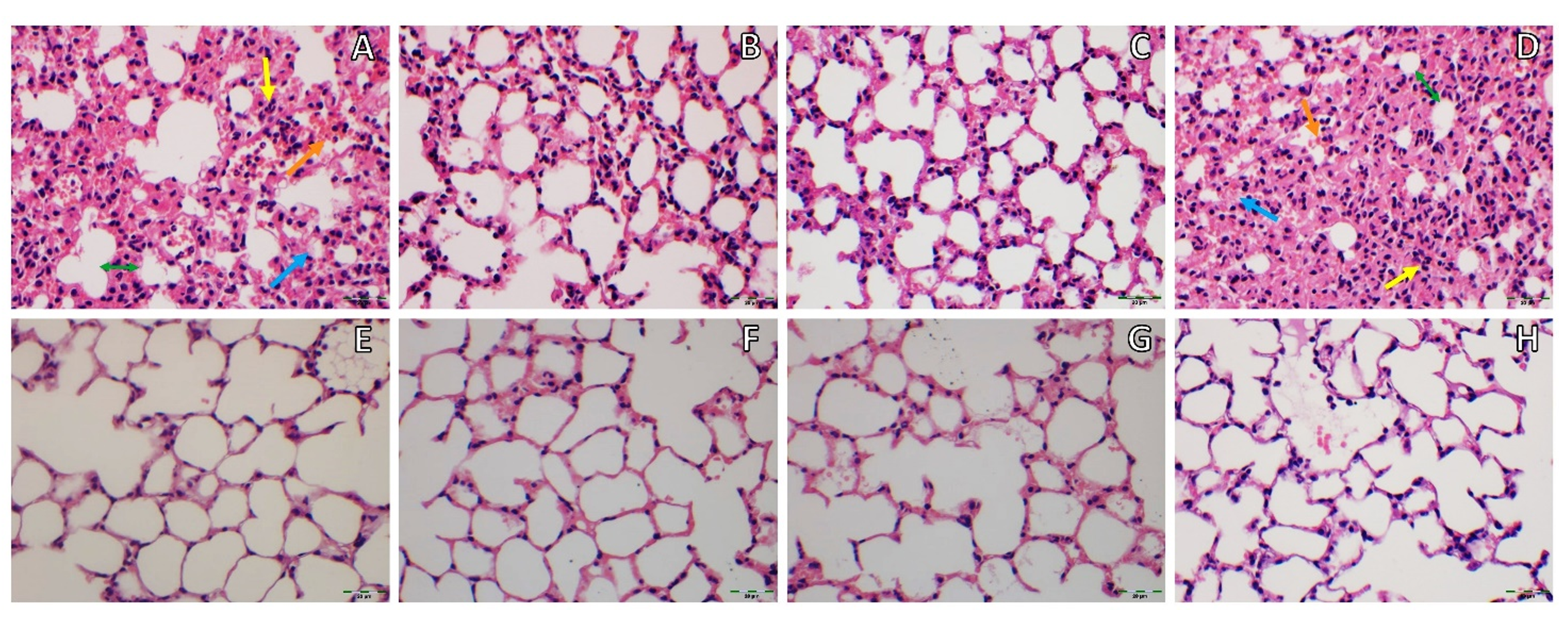

Supplement: Supplementary file 1 [file supplementary_material.zip › Fig_S6.tif]

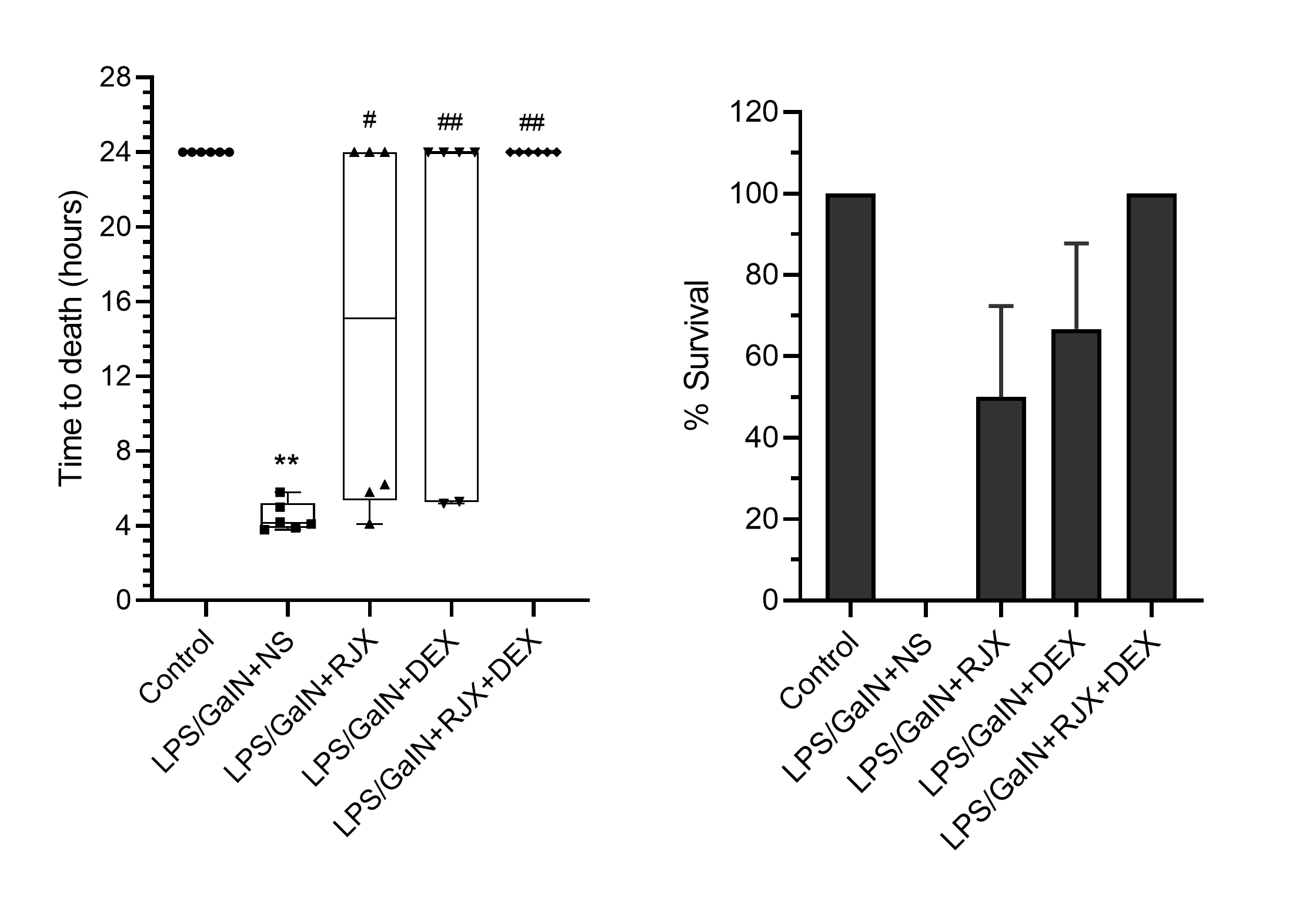

Supplement: Supplementary file 1 [file supplementary_material.zip › Fig_S7.tif]

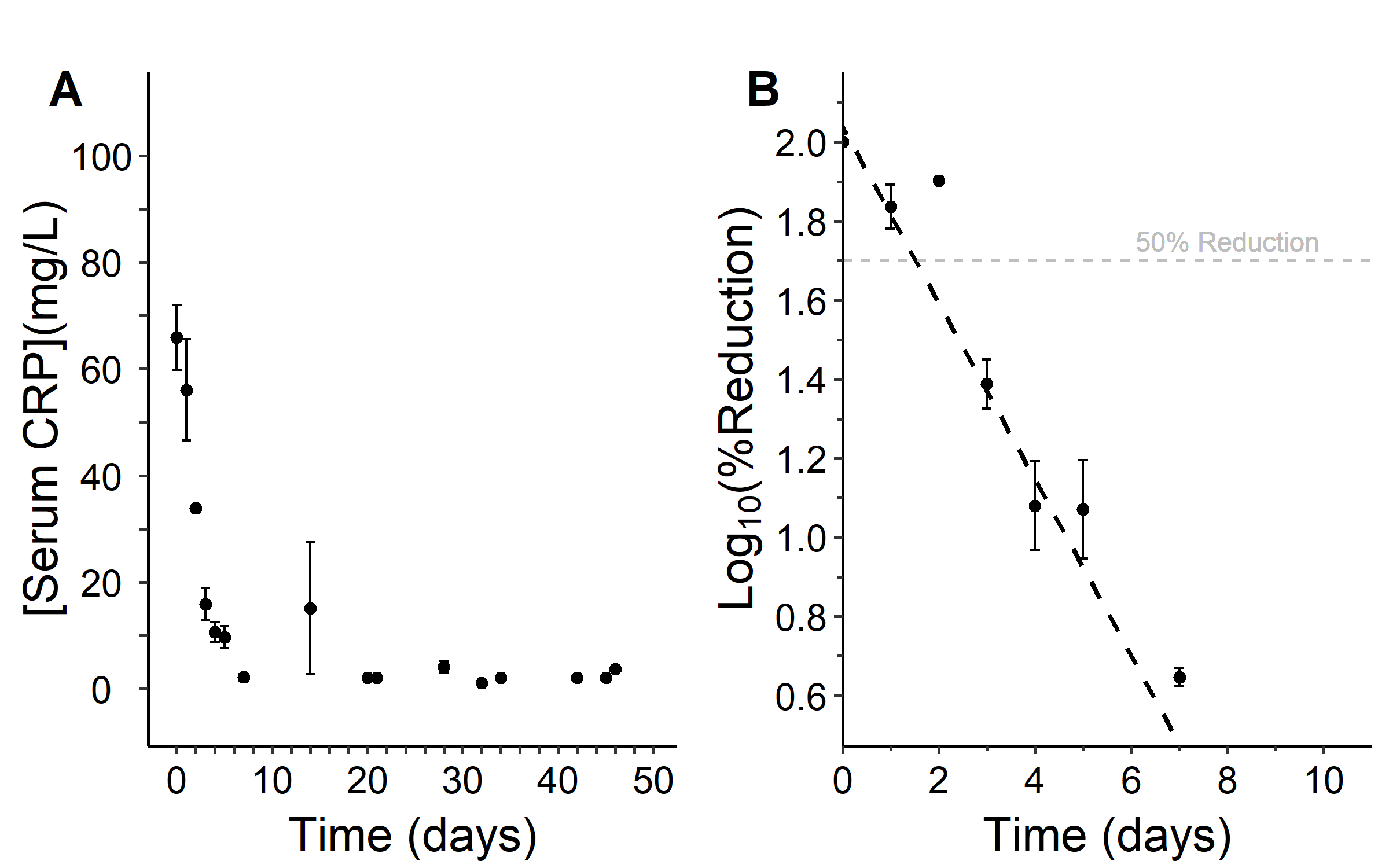

Supplement: Supplementary file 1 [file supplementary_material.zip › Fig_S8_Serum_CRP_kinetic_rev2.tiff]

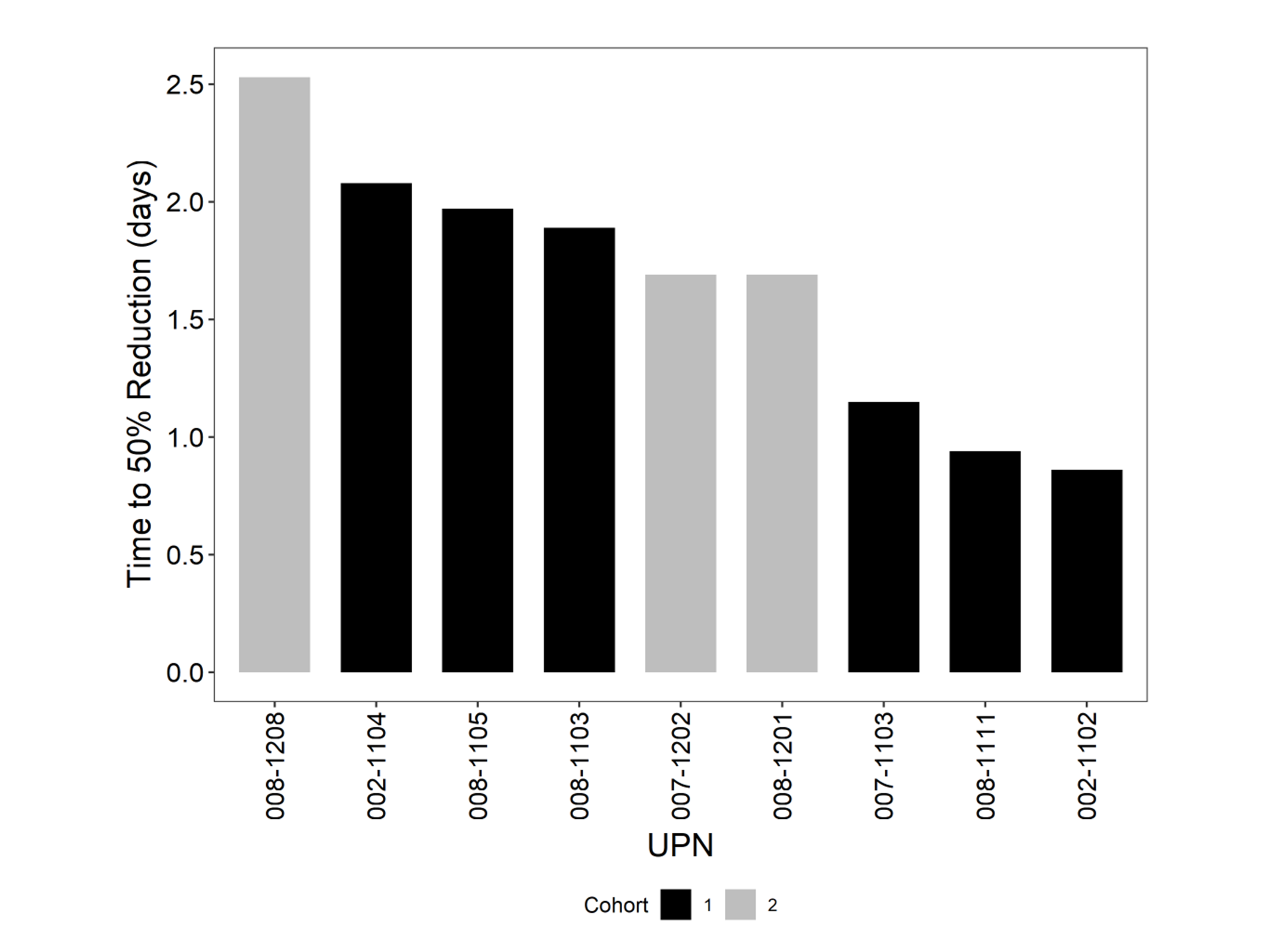

Supplement: Supplementary file 1 [file supplementary_material.zip › Fig_S9_Time_50.tif]
